# Techno-economic Assessment of CO_2_ Electrolysis: How Interdependencies between Model Variables Propagate Across Different Modeling Scales

**DOI:** 10.1021/acssuschemeng.3c02226

**Published:** 2023-06-28

**Authors:** Isabell Bagemihl, Lucas Cammann, Mar Pérez-Fortes, Volkert van Steijn, J. Ruud van Ommen

**Affiliations:** † Department of Chemical Engineering, Delft University of Technology, Van der Maasweg 9, 2629 HZ Delft, The Netherlands; ‡ Department of Engineering Systems and Services, Delft University of Technology, Jaffalaan 5, 2628 BX Delft, The Netherlands

**Keywords:** CO_2_ electrolysis, First-principle electrolyser model, Multiscale modeling, Optimization, Techno-economic analysis

## Abstract

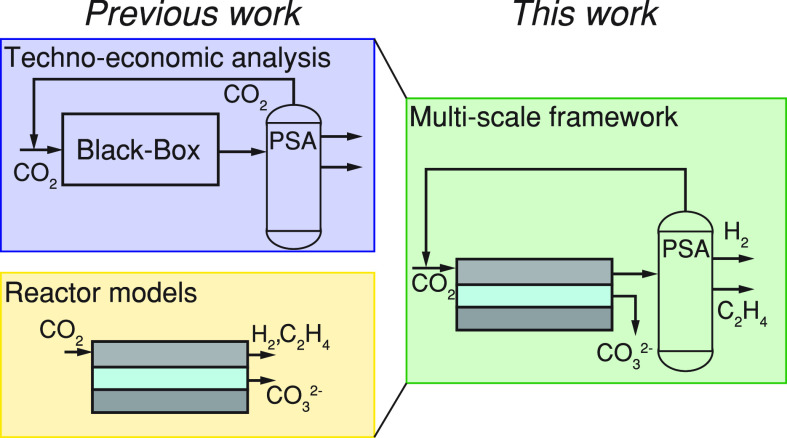

The production of base chemicals by electrochemical conversion of captured CO_2_ has the potential to close the carbon cycle, thereby contributing to a future energy transition. With the feasibility of low-temperature electrochemical CO_2_ conversion demonstrated at lab scale, research is shifting toward optimizing electrolyser design and operation for industrial applications, with target values based on techno-economic analysis. However, current techno-economic analyses often neglect experimentally reported interdependencies of key performance variables such as the current density, the faradaic efficiency, and the conversion. Aiming to understand the impact of these interdependencies on the economic outlook, we develop a model capturing mass transfer effects over the channel length for an alkaline, membrane electrolyser. Coupling the channel scale with the higher level process scale and embedding this multiscale model in an economic framework allows us to analyze the economic trade-off between the performance variables. Our analysis shows that the derived target values for the performance variables strongly depend on the interdependencies described in the channel scale model. Our analysis also suggests that economically optimal current densities can be as low as half of the previously reported benchmarks. More generally, our work highlights the need to move toward multiscale models, especially in the field of CO_2_ electrolysis, to effectively elucidate current bottlenecks in the quest toward economically compelling system designs.

## Abstract 2

Coupling the analysis of the technical feasibility with the economic evaluation for electrochemical CO_2_ conversion processes allows to identify relevant targets for technology development.

## Introduction

**Figure 1:**
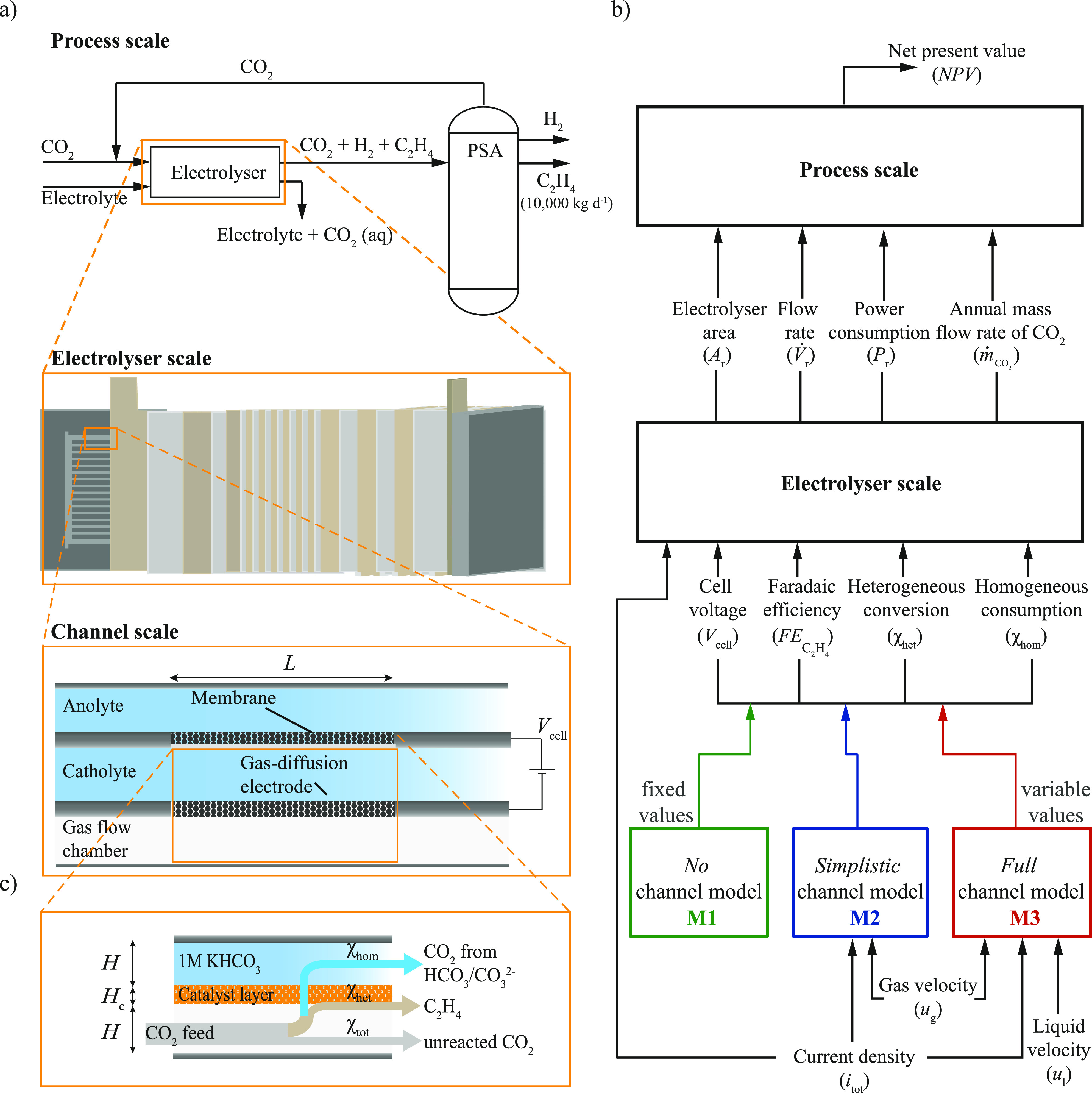
Overview of the connection between the process, electrolyser, and channel scale model (a) with the relevant in- and output variables and parameters to couple the scales (b). The *no* channel model (M1) uses fixed performance variables, while the *simplistic* channel model (M2) and the *full* channel model (M3) capture the different pathways of CO_2_ through the electrolyser (c) with increasing level of detail.

The current anthropogenic carbon economy does not possess the ability to reduce CO_2_. Instead, it solely oxidizes various fossil-based carbon sources to CO_2_, leading to increasing atmospheric concentrations. Closing the carbon cycle by converting waste CO_2_ to bulk chemicals is a promising avenue to minimize emissions and fossil-based resource consumption.^1,2^ One technology offering the potential of achieving this transition is the electrochemical conversion of CO_2_.^3,4^ Techno-economic studies have led researchers to identify target values for performance variables^5−7^ and pathways toward the profitable deployment of this emerging technology.^8−12^ The first techno-economic analyses studied the economic feasibility of electrochemical CO_2_ reduction by presenting target values for the performance variables to reach a break-even point.^5,6^ These performance variables include the current density, the faradaic efficiency, and the cell potential, while the conversion rate is fixed.^5,6,13,14^ Importantly, these variables are usually assumed to be independent.^5−7,13,15^ Under this assumption, the threshold values for the first three variables were derived utilizing a generalized electrochemical CO_2_ reduction plant model based on a fixed conversion rate and price indication for existing electrolyser technologies.^5,6,13^ The derived thresholds include current densities above 250–300 mA cm^–2^ and cell potentials below 1.8 V to reduce capital and operational costs of the electrolyser unit, respectively.^6,13^ Further, faradaic efficiencies above 80–90% reduce downstream separation costs of the product.^6,13^ Although these thresholds provide significant guidance for experimental studies, their underlying analyses neglect the interdependencies of current density, faradaic efficiency, cell potential, and conversion on the mechanistic level.^11,16^ This confines the techno-economic analyses to univariate sensitivity analyses, potentially leading to overestimation of the solution space for feasible performance values and electrolyser designs. Understanding the interdependencies of the performance variables is thus crucial for electrolyser design, operation, and optimization. Therefore, experimental studies on electrolyser design have mostly been accompanied by modeling efforts, to capture the interdependencies at the channel scale.^17−24^ These models are used to resolve local effects in the electrolyser, for example, to understand concentration gradients and mass transfer limitations due to the change in pH near the catalyst layer.^17,19^ While these models can provide relevant insights into the interdependencies of the performance variables, they so far have not been translated into techno-economic analyses. Channel models can additionally account for concentration gradients along the flow channel, taking into account their effect on single-pass conversion.^25−27^ For example, the study of Kas et al.^25^ showed an increased loss of CO_2_ to carbonate formation at high current densities due to the limited buffer capacities of the electrolyte. This insight reveals a trade-off between current density and conversion, one of the interdependencies commonly neglected when using fixed performance variables for techno-economic analyses. This study presents a multiscale modeling approach ranging from the mechanistic channel scale over the electrolyser stack scale to the process scale (Figure 1a), assessing interdependencies on the electrolyser design level from an economic perspective. For the multiscale model, a channel model accurate enough to capture interdependencies between the performance metrics of the electrolyser is developed, which then allows evaluation and demonstration of the influence of the interdependencies on the selected process economic indicator. The channel model is based on a first-principle model of an alkaline flow-through CO_2_ electrolyser for the production of ethylene, capturing the interplay between CO_2_ conversion, faradaic efficiency, and cell voltage for varying current densities. This interplay, in turn, influences the electrolyser and downstream unit investment and operating costs. Employing this multiscale framework for techno-economic assessment and optimization allows for computing the desired target performance variables based on mechanistic insights.

## Multiscale Model

The multiscale modeling framework comprises three scales: the process, the electrolyser, and the channel scale, as shown in Figure 1a. While the multiscale model is generic and independent of the desired product, we will show the model setup and results for ethylene as the main product with a target production rate of 10,000 kg d^–1^. The choice of this gaseous throughput is motivated by the objective to investigate industrially relevant conditions while ensuring that the financial correlations used for the cost estimate remain applicable. The throughput is used to calculate investment and operating costs for the electrolyser and gas separation, herein considered to be a pressure-swing adsorption unit (PSA). The design of the electrolyser is based on a flow-through gas diffusion electrode (GDE) cell, motivated by its extensive application in experimental studies aiming for high current densities.^20,28,29^ The electrolyser is operated under ambient pressure and room temperature (see Table 1). The liquid catholyte flow rate is fixed to evaluate the electrolyser performance, while the liquid postprocessing is not considered in the cost evaluation. It is assumed in the model that the electrolyser is continuously fed with fresh electrolyte and all formed ionic species, for example bicarbonate, leave the reactor with the liquid electrolyte stream. The gas phase is solely composed of the reactant CO_2_, the target product C_2_H_4_, and the side product H_2_, as shown in the flowchart in Figure 1 a).

**Table 1:** Overview of Channel Dimensions and Operating Parameters

Parameter	Unit	Value	Description
*L*	[m]	0.10	Channel length
*H*	[m]	1.00 × 10^–3^	Channel height
*W*	[m]	1.00 × 10^–2^	Channel width
*H*_c_	[m]	3.00 × 10^–6^	Catalyst layer thickness
ϵ	[-]	0.70	Porosity
*T*	[K]	300	Temperature
*P*	[Pa]	1.00 × 10^5^	Pressure

**Figure 2:**
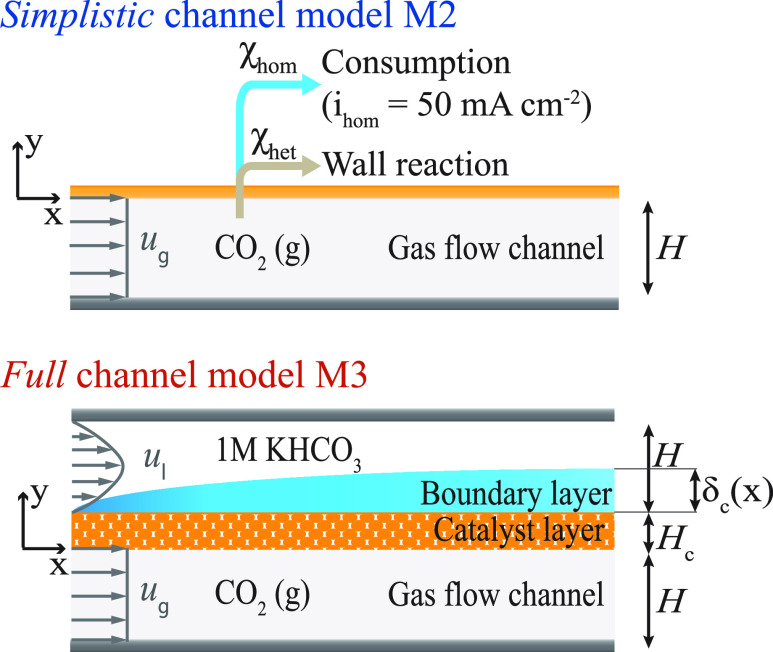
*Simplistic* channel model (M2) with an assumed, fixed homogeneous consumption of CO_2_, and the *full* channel model (M3) with a fully resolved homogeneous consumption of CO_2_.

The electrolyser performance is described based on the following five performance variables (Figure 1b): the current density *i*_tot_, the cell voltage *V*_cell_, the faradaic efficiency *FE*_C_2_H_4__, the heterogeneous conversion χ_het_, and the homogeneous consumption χ_hom_. The energy efficiency is not considered separately in this work as it directly follows from the cell voltage and faradaic efficiency.^13^ While the first three are common terms in electrochemistry, the last two are understood as the conversion rates of (i) CO_2_ due to the heterogeneous electrochemical reaction at the catalyst forming the reaction product (often referred to as single pass conversion^29−31^) and (ii) the loss of CO_2_ due to the homogeneous carbon equilibrium reactions in the liquid electrolyte, respectively (Figure 1c). The current state of the art techno-economic assessments rely on a fixed set of these performance variables, which are chosen independently of each other.^5,6,11,13^ To illustrate the propagation of the interdependencies across scales, we introduce three exemplary models (see Table 2). The *no* channel model (M1) is based on the current state of the art and, therefore, neglects the interdependencies on the channel scale. For M1, we use a variable current density *i*_tot_ = [50–250 mA cm^–2^] in combination with fixed electrochemical variables *V*_cell_ = 3.69 V and *FE*_C_2_H_4__ = 0.7, and the fixed conversion rates χ_het_ = 0.5 and χ_hom_ = 0. The *simplistic* (M2) and *full* (M3) channel models are governed by the physics at the channel scale with increasing level of detail (Figure 2). They, therefore, capture interdependencies between the performance variables as further explained in the following section. All three models are embedded in the same electrolyser and process scale model.

**Table 2:** Values of Performance Variables for the *No* (M1), *Simplistic* (M2), and *Full* (M3) Channel Model, with a Variable Current Density *i*_tot_ = [50–250 mA cm^–2^]^a^

Model	*V*_cell_ [V]	*FE*_C_2_H_4__ [-]	χ_het_ [-]	χ_hom_ [-]
M1	3.69	0.70^28^	0.50^6^	0.00^6^
M2	*f*(*i*_tot_)	*f*(···)	*f*(···)	0.13^b^
M3	*f*(*i*_tot_)	*f*(···)	*f*(···)	*f*(···)

a Performance variables which are dependent on more than one other variable are denoted as *f*(···).

b With a fixed additional current density (*i*_hom_ = 50 mA cm^–2^) and a single channel gas flow rate of 10 sccm, for more details see section S1.

### Channel Scale Model

We consider two-dimensional channel scale models for a GDE-cell, which are most applicable for shallow channels in which the height is much smaller than the width (*W* = 10*H* in this work; Table 1). The interdependencies among the performance variables *i*_tot_, *V*_cell_, *FE*_C_2_H_4__, χ_het_, and χ_hom_ are captured through a mechanistic model of the GDE-cell. The GDE-cell is characterized by a gaseous and a liquid flow channel separated by a gas diffusion electrode in the cathode compartment. The anode is separated from the cathode side by a proton exchange membrane (Figure 1 a), which is not explicitly modeled in this work.

The *simplistic* channel model (M2) solely considers the gas flow channel, with the catalyst layer modeled as an abrupt interface (see Figure 2, top). For this, the description of the homogeneous consumption (χ_hom_) is fixed and solely depends on the single channel gas flow rate *u*_g_ (see section S1). The *full* channel model (M3) considers, in addition to the gas flow channel, the catalyst and the liquid boundary layer in the electrolyte chamber (see Figure 2, bottom). Model M3 hence includes the parasitic homogeneous reactions occurring in the catalyst layer, thereby allowing to fully resolve the homogeneous consumption. The governing equations for M2 and M3 are given in the following sections, together with the relevant assumptions. The chosen channel dimensions and operating parameters are listed in Table 1. All other input parameters used in models M2 and M3 are listed in section S2. All relevant derivations, boundary conditions, the model validation, and the discussion of assumptions for model M3 are given in section S3.

#### Mass Transport and Species Balance

We present a model to describe the concentration *c*_k_ of species k along the channel length and across the three layers (gas flow channel, catalyst layer, and liquid boundary layer) with coordinates *x* and *y* as defined in Figure 2. The *simplistic* channel model M2 and the *full* channel model M3 share the same modeling domain for the gas flow channel. In the gas flow channel, pure gaseous CO_2_ is introduced and described by plug-flow behavior. The gaseous mass transport in the porous gas diffusion layer is neglected and the catalyst layer is assumed to be fully flooded. Therefore, the phase transfer of gaseous CO_2_ to the liquid electrolyte occurs at the interface of the gas channel and the catalyst layer. The concentration of CO_2_ is assumed to be in equilibrium at the gas–liquid interface. The species balance for gaseous compounds (CO_2_(*g*), C_2_H_4_(*g*), and H_2_(*g*)) in the gas channel is described by
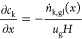
1where *u*_g_ is the superficial gas velocity in the gas flow channel and *H* is the channel height (see section S3.1 for derivation). The term *ṅ*_k,gl_(*x*) denotes the molar flux (per unit area) across the gas-catalyst interface. This flux is equal to the molar production or consumption rate of the gaseous compounds over the catalyst layer height *H*_c_ at any location *x* in the single channel
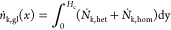
2The term *Ṅ*_k,het_ denotes the consumption/production rate of CO_2_(*g*), C_2_H_4_(*g*), and H_2_(*g*) in mol s^–1^ m^–3^ due to the heterogeneous electrochemical reactions, while *Ṅ*_k,hom_ in eq 2 denotes the consumption rate of the dissolved CO_2_(*aq*) due to the homogeneous buffer reactions in the liquid electrolyte.

For the *simplistic* channel model (M2) the catalyst layer is not explicitly modeled, with the consumption rate considered as part of the heterogeneous reaction term, adding an additional current density for the homogeneous consumption rate of *i*_hom_ = 50 mA cm^–2^ (see section S1).^11,26^ By approximating the homogeneous consumption with a fixed additional heterogeneous reaction rate, the species balance for dissolved CO_2_(*aq*) and the ionic species does not need to be solved. This approach eliminates *Ṅ*_k,hom_ from eq 2 and therefore allows straightforward calculation of the (single channel gas flow rate dependent) consumption rate (section S1). The concentration of the gaseous compounds CO_2_, C_2_H_4_, and H_2_ along the channel length is then fully described by eqs 1 and 2.

For the *full* channel model (M3), the catalyst layer and the liquid boundary layer are fully captured by explicitly solving the species balance for all species (including OH^–^, HCO_3_^–^, and CO_3_^2–^ as the ionic species considered in this work), which allows one to calculate the homogeneous consumption rate. Similar to previous modeling studies^19,25^ in the following we neglect migration for all ionic species and the crossover of carbonate and bicarbonate to the anode side.^32−34^ The steady state species balance of the dissolved CO_2_(*aq*) and the ionic species in the catalyst layer (0 ≤ *y* < *H*_c_) is then governed by diffusion as well as homogeneous and heterogeneous reactions^19^
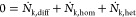
3with the term *Ṅ*_k,diff_ accounting for species transport through diffusion. In the catalyst layer, this term is calculated via
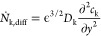
4with the diffusion coefficient *D*_k_ and the prefactor arising from the porosity ϵ and tortuosity τ = ϵ^–1/2^.^35^ Outside the catalyst layer, within the boundary layer (*H*_c_ < *y* < *H*_c_ + δ_c_(*x*)), the balance equation of the dissolved CO_2_(*aq*) and the ionic species is only governed by diffusion^25^
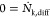
5with the diffusive transport given as
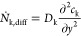
6

The formation of the diffusive boundary layer on the liquid electrolyte side hinders the transport of fresh electrolyte to the catalyst layer, resulting in an increase in local pH and homogeneous reaction rate in the catalyst layer along the channel length.^25^ This effect is included by calculating the thickness of the boundary layer according to the Lévêque approximation^36^
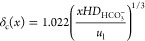
7with the average liquid electrolyte velocity *u*_l_. The Lévêque approximation entails that two assumptions need to hold for eq 7 to be a good approximation: (a) constant concentration at the catalyst–electrolyte interface and (b) a developing boundary layer with δ_c_(*x*) ≪ *H*.^37^ Since the supply of HCO_3_^–^ is the limiting factor in retaining the electrolyte buffer capacity in the catalyst layer,^25^ the length of the boundary layer is equally calculated for all species using the diffusion coefficient *D*_HCO_3_^–^_. The concentrations of the ionic species are fixed to the equilibrium concentration in the 1 M KHCO_3_ CO_2_ saturated electrolyte at the liquid electrolyte/boundary layer interface, and the no flux boundary condition is imposed at the catalyst/gas channel interface. Similarly, the concentration of the dissolved CO_2_(*aq*) is assumed to be in equilibrium with the gaseous CO_2_(*g*) concentration at the catalyst/gas interface, and the no flux boundary condition is imposed at the catalyst/boundary layer interface (see section S3.2). This allows the species balance in the catalyst (eq 3) and boundary layer (eq 5) to be calculated, which are coupled through eq 2 to the species balance in the gas channel (eq 1). The concentration profile in the gas channel, catalyst layer, and liquid boundary layer is thereby fully described, with calculations of the required heterogeneous production and homogeneous consumption rate given in the following sections.

#### Heterogeneous Reactions

The heterogeneous electrochemical reduction reactions of CO_2_(*aq*) and H_2_O(*l*) occur in the catalyst layer. Copper catalysts form a wide distribution of gaseous and liquid products, which in this work are limited to C_2_H_4_ and H_2_, by considering the following two cathodic reactions:
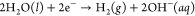
8
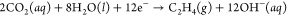
9Note that this is a simplification in this work, and that, to the best of the authors knowledge, no catalyst for selective ethylene production has been reported. At the anode, the oxygen evolution reaction is facilitated, i.e.
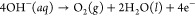
10The electrochemical reaction rate for the species consumed or formed at the electrodes is calculated via Faraday’s law^38^
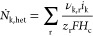
11in which ν_k,r_ denotes the stoichiometric coefficient for species k in reaction r, *z*_r_ the amount of transferred electrons in reaction r, and *F* the Faraday constant. The current density *i*_tot_ is calculated via the Tafel equation fitted to experimental data reported by Tan et al.^29^ (see section S3.3)
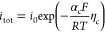
12with *i*_0_ the exchange current density, α_c_ the transfer coefficient, *R* the universal gas constant, and η_c_ the applied cathode overpotential. In fitting the data for the current density, *i*_tot_, all reported carbonaceous species are considered to be ethylene, thereby simplifying the kinetic expression. It is further assumed that hydrogen is only produced at the onset of mass transport limitations toward CO_2_,^39,40^ which ensures that the current density *i*_tot_ remains constant over the electrode length (galvanostatically controlled) leading to the following partial current densities of C_2_H_4_ and H_2_:
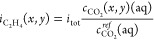
13
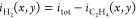
14with *c*_CO_2__^*ref*^(*aq*) the CO_2_ equilibrium concentration within the electrolyte at standard conditions (*P* = 1.00 × 10^5^ Pa and *T* = 300 K). The dissolved CO_2_(*aq*) concentration in the liquid electrolyte (*c*_CO_2__(*x*, *y*)(*aq*)) relates to the gaseous CO_2_ concentration along the channel length *c*_CO_2__ through Henry’s law. The changes in the CO_2_(*aq*) concentration over the catalyst layer height are driven by the heterogeneous consumption (eq 11) and homogeneous conversion (eq 17). The motivation and limits of these simplified kinetics are discussed in section S3.4.

#### Homogeneous Reactions

In addition to the heterogeneous reaction, CO_2_ is also consumed by homogeneous reactions within the electrolyte in the catalyst layer. These reactions are constituted by the bicarbonate-buffer reactions, balancing the pH of the solution. This reaction mechanism is only considered in model M3 and described by the following equilibrium reactions^19^
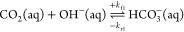
15
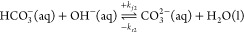
16where *k*_f1_ and *k*_f2_ are the forward reaction rate constants and *k*_r1_ and *k*_r2_ the respective reverse reaction rate constants, the values to all of which are provided in Table S4. Under consideration of the above equilibrium reactions, the volumetric homogeneous reaction terms can be written as^17^
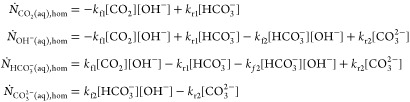
17with the notation [*c*] used for the concentration *c*_k_. The significance of the homogeneous reactions can be explained by the increased rate of eq 15 at higher alkalinity, which inevitably occurs at elevated heterogeneous reaction rates due to increased hydroxide production. For simplicity, it is assumed that the homogeneous reactions occur solely within the catalyst layer, where the pH and CO_2_(*aq*) concentration are highest. The void fraction within the catalyst layer is not accounted for.

### Electrolyser Scale Model

The electrolyser scale model couples the calculated concentration profiles from the channel scale model to the process scale model (Figure 1b). First, the input variables χ_het_, χ_hom_, *FE*_C_2_H_4__, and *V*_cell_ are calculated based on the C*O*_2_(*g*) and C_2_*H*_4_(*g*) concentrations obtained from the channel scale models M2 and M3 for a variable *i*_tot_. For this, the electrolyser is assumed to be composed of a number of hydraulically, thermally, and electrically independent channels. The overall conversion achieved in the electrolyser then equals the single-channel conversion, which is calculated assuming a constant pressure as
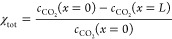
18with the channel length *L*. The heterogeneous conversion is calculated as^29−31^
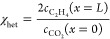
19with 2 being the stoichiometric coefficient (see eq 9). The homogeneous consumption is then calculated as the difference between those figures, i.e.
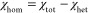
20The faradaic efficiency (selectivity) toward ethylene is calculated based on the product concentration and gas velocity *u*_g_ at the channel outlet^32^ (see section S4)
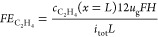
21with 12 being the amount of electrons required to reduce CO_2_ to C_2_H_4_. The cell voltage is further related to the current density (see eq 12) via
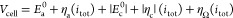
22with the constant anodic and cathodic standard potentials *E*_a,c_^0^ and the current density dependent overpotentials η_a,c,Ω_ (see section S5 for more details). The input variables for the electrolyser scale for models M2 and M3 (Table 2) are then fully described by eqs 18–22.

Based on these input variables, the required electrolyser area *A*_r_, the volumetric gas flow rate *V̇*_r_, the annual mass flow rate *ṁ*_CO_2__, and the power consumption *P*_r_ are calculated next (see section S4 for derivations), to estimate the investment and operating costs for the electrolyser and separation unit. The required electrolyser area is calculated taking into account the faradaic efficiency as well as the current density
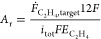
23with the daily production target 10,000 kg d^–1^ converted to *Ḟ*_C_2_H_4_,target_ ≈ 4.13 mol s^–1^. Further, the volumetric flow rate associated with the electrolyser setup is calculated from the heterogeneous conversion and target production rate as
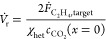
24The annual mass flow rate of CO_2_ through the electrolyser then follows from the annual production target (*ṁ*_C_2_H_4_,target_ = 3,500,000 kg yr^–1^). The corresponding annual consumption rate of CO_2_ equals
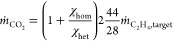
25Finally, the overall power consumption *P*_r_ follows from the product of the cell voltage and current density, i.e.
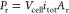
26

### Process Scale Model

The process scale model describes the overall process and its economic performance. This model represents a simplified plant layout based on previous techno-economic analyses,^6,13^ with a CO_2_ feed source through direct air capture (DAC), an electrolyser unit for the electrochemical CO_2_-reduction, and subsequent gas separation in a pressure swing adsorption (PSA) unit (Figure 1 a)). Liquid pre- and postprocessing steps are not taken into account. The currency used is the US dollar (multiple years).

The selected process economic indicator is the end-of-lifetime net present value (*NPV*) of the overall process, assuming 20 years of continuous operation.^6^ The *NPV* is calculated by taking into account the cash flow *CF*(*t*) on an annual basis as
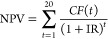
27in which *t* is the respective year of operation. The term *IR* denotes the interest rate and is assumed to be 10% throughout the lifetime.^6^ It is assumed that the plant is erected within the first year and operates at full capacity for the remaining 19 years of operation with the cash flow calculated as
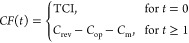
28where *C*_rev_, *C*_op_, and *C*_m_ describe the annual revenue, operating costs, and maintenance costs, respectively. TCI is the total capital investment, which comprises the costs for the electrolyser, the separation unit, and all additional infrastructural facilities (balance of plant). The investment costs for the electrolyser are proportional to the required electrolyser area ($920 m^–2^),^6^ while the costs for the PSA unit scale with the overall volumetric flow rate at the electrolyser outlet with a reference cost^41^ of 1.99 M$ and a reference flow rate of 1000 m^3^ h^–1^. The balance of plant costs are assumed to make up 35/65 of the electrolyser costs.^6^ The total capital investment based on the equipment costs can then be calculated as
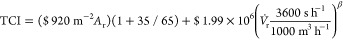
29where *A*_r_ is the required area (eq 23) and *V̇*_r_ the volumetric flow rate at the electrolyser outlet (eq 24). The term β is a fitting factor associated with the cost correlation for the PSA unit, assumed to be 0.7 according to the regression function proposed by Paturska et al.^41^ for flow rates between 500 m^3^ h^–1^ and 1400 m^3^ h^–1^.

The annual revenue depends on the annual production target, and market price of ethylene (herein taken as $1.3 kg^–1^)^6^ as
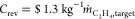
30The annual costs are then determined by the CO_2_ price (herein taken as $0.04 kg^–1^),^13^ which is slightly lower than the most optimistic assumption for commercial DAC units using chemical absorption.^42^ For the electricity price, an optimistic value of $0.03 kWh^–1^ is taken based on predictions published by Haegel et al.^43^ The operating costs associated with separation are assumed to be only made up of the electricity costs (0.25 kW h m^–3^),^41^ which allows calculating the overall operating costs based on the annual consumption rate of CO_2_ (eq 25), the overall power consumption (eq 26), and the volumetric flow rate (eq 24) as
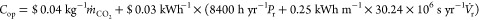
31The annual maintenance costs are taken to be 2.5% of the capital investment costs for the electrolyser,^6^ i.e.
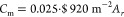
32As a comparative figure for the models (M1 to M3) the *relative NPV* is defined as follows
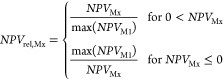
33for any model M_*x*_ with x ∈ [1, 2, 3] (for further detail, see section S6.1). The highest *NPV* of model M1 is taken as a reference point, as at this value the investment costs for the electrolyser do not influence the overall *NPV* anymore and the resulting current density is commonly reported as the target value in techno-economic analyses.^5,6,13^

### Implementation

For the *no* channel model M1 the input variables to the electrolyser scale are fixed (Table 2) such that the required electrolyser area, the volumetric gas flow rate, the annual mass flow rate of CO_2_, and the overall power input required to achieve the target production for C_2_H_4_ (eqs 23–26) can be straightforwardly calculated. The *relative NPV* (eq 33) is then calculated based on these variables as a comparison metric. For the *simplistic* channel model (M2) and the *full* channel model (M3), the input variables to the electrolyser scale depend on the output of the channel scale. Therefore, the concentrations of CO_2_, C_2_H_4_, and H_2_ along the channel length need to be calculated first. The concentrations are then used to calculate the input variables to the electrolyser scale (eqs 18–22) and subsequently the input variables to the process scale are calculated (eqs 23–26), followed by the *NPV* and *relative NPV*.

The species balance (eq 1) in the gas channel is solved for models M2 and M3 via Heun’s method for varying current densities (*i*_tot_) and gas velocities (*u*_g_). For the *simplistic* model (M2), the flux across the gas-catalyst interface (eq 2) is given as a boundary condition, while for the *full* channel model (M3) the flux across the gas-catalyst interface is updated at every finite difference by solving the governing equations in the catalyst and boundary layer (eq 3 and 5). The solution to these respective domains is found with the Matlab R2020a built-in solver bvp4c, where the extent of the boundary layer is adapted on each step according to eq 7 and the solution of the previous step is supplied as an initial guess to the current step. The extend of the boundary layer is calculated for a fixed electrolyte flow rate of 0.54 m s^–1^ to minimize the consumption of CO_2_ due to the buffer reaction,^25^ while still ensuring operation in the laminar flow regime.

To gain insight into the optimal mode of operation, the model has been constructed to allow for facile use with the Matlab built-in nonlinear optimizer fmincon and fminsearch. All Matlab scripts are made available via GitHub (see Data Availability).

## Results and Discussion

In this section, we first discuss the interdependencies between three performance variables on the channel scale for the *no* channel (M1), the *simplistic* channel (M2), and the *full* channel (M3) model. For this, we consider the heterogeneous conversion χ_het_, the homogeneous consumption χ_hom_, and the faradaic efficiency *FE*_C_2_H_4__ and their dependence on the current density. As the cell voltage *V*_cell_ can be straightforwardly calculated (eq 22) it is not explicitly discussed. Second, we discuss the propagation of the interdependencies from the channel scale to the process scale model in terms of the *relative NPV* for varying current densities and gas velocities. The contribution of selected technical input variables to the process *NPV*, together with the contribution of economic parameters, is evaluated in a sensitivity analysis in Section S6.2. Lastly, the optimization results are presented and discussed in light of current developments in the literature.

### Interdependency of Performance Variables on the Channel Scale

**Figure 3:**
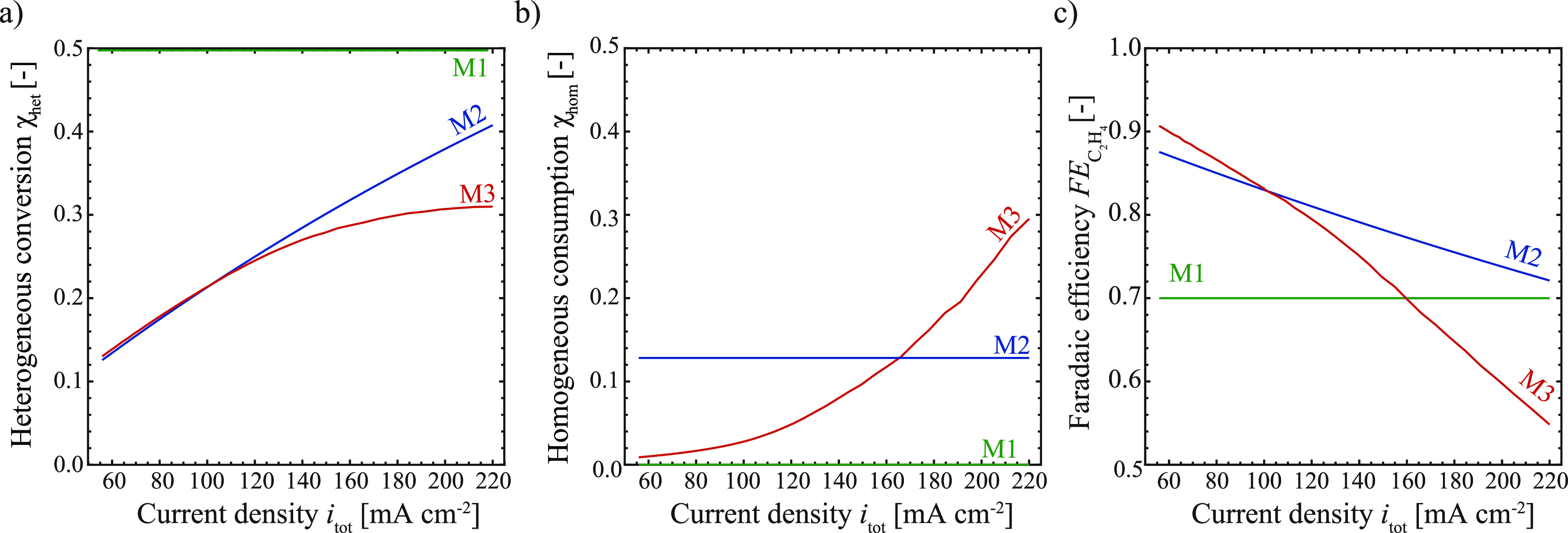
Heterogeneous conversion (a), homogeneous consumption (b), and faradaic efficiency (c) for models M1 to M3 as a function of current density, with a fixed channel geometry (Table 1) and a single channel gas flow rate of 10 sccm (*u*_g_ = 0.0167 m s^–1^). For model M2, a fixed additional current density (*i*_hom_ = 50 mA cm^–2^) is used to account for the homogeneous reactions, while these are fully resolved in model M3 for a fixed single channel liquid flow rate of 325 mL min^–1^ (*u*_l_ = 0.54 m s^–1^).

The heterogeneous conversion (χ_het_), homogeneous consumption (χ_hom_), and selectivity (*FE*_C_2_H_4__) with varying current densities (*i*_tot_) are presented for models M1 to M3 in Figure 3. Contrary to the case of fixed performance variables (M1), using a mechanistic channel scale model results in a dependency of the above-stated variables predicting the expected trend of increased conversion rates with higher current densities as shown in Figure 3a. For low conversions, this trend is linear, as the supply of CO_2_ to the catalyst is not limited (for more details, see section S7). For the *simplistic* channel model M2, the limit of the linear scaling is reached at higher conversions than that for the *full* channel model (M3). This can be explained by comparing the trends for the consumption rate of CO_2_ for models M2 and M3 in Figure 3b. While the consumption rate is constant in model M2 the consumption of CO_2_ in M3 increases with increasing current densities. Increased consumption rates of CO_2_ limit the availability of CO_2_ at the catalyst site and therefore limit the heterogeneous conversion, leading to a deviation from the linear scaling at lower current densities for M3 compared to M2. The steep exponential increase in homogeneous consumption for model M3 can be explained by the dependence of the formation of hydroxide ions on the current density, as seen in eqs 8 and 9, paired with the limited buffer capacity of the electrolyte. This eventually leads to higher consumption rates through the homogeneous reaction than through the heterogeneous reaction at high current densities. This, in turn, influences the selectivity, resulting in a steep decrease of *FE*_*C*_2_H_4__ toward the formation of ethylene with increased current density for M3 as shown in Figure 3c. For a constant consumption rate in M2 the faradaic efficiency toward ethylene displays a weaker dependency on the current density because the main driver for the depletion of CO_2_ at high current densities is the increased heterogeneous conversion instead of the homogeneous consumption.

### Propagation of Interdependencies from the Channel Scale to the Process Scale Model

**Figure 4:**
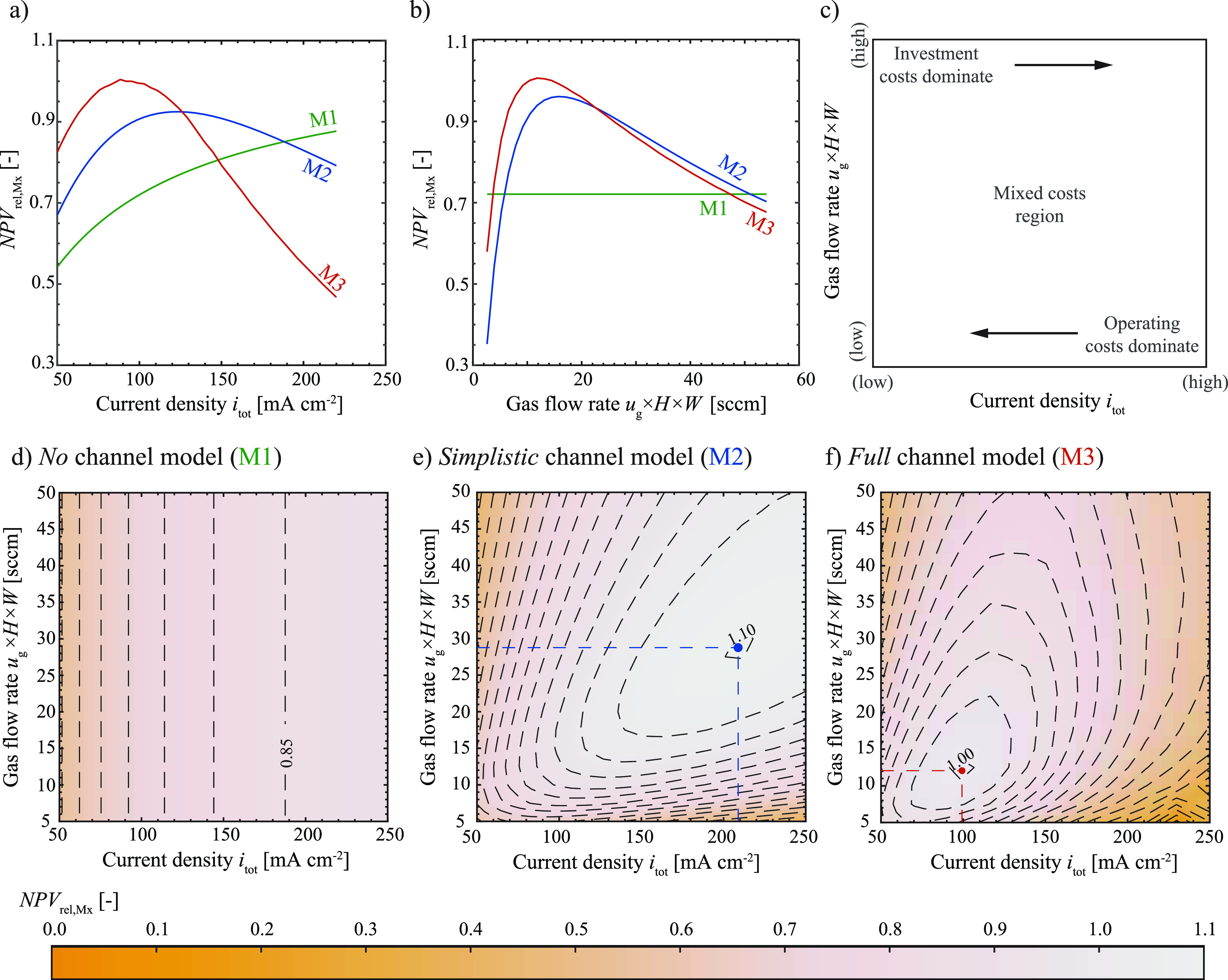
*Relative NPV* as a function of current density for a fixed single channel gas flow rate *u*_g_ × *H* × *W* = 10 sccm (a) and as a function of the single channel gas flow rate for a fixed current density *i*_tot_ = 100 mA cm^–2^ (b). Schematic of main cost drivers for varying current densities and single channel gas flow rates with the arrows indicating the direction of decrease of the respective cost unit (c). Contours (with an equidistant spacing of 0.05) of the *relative NPV* for varying current densities and single channel gas flow rates for M1 (d), M2 (e), and M3 (f) with the dots indicating the optimum for model M2 and M3. For model M2 a fixed additional current density (*i*_hom_ = 50 mA cm^–2^) is used to account for the homogeneous reactions, while these are fully resolved in model M3 for a fixed single channel liquid flow rate of 325 mL min^–1^ (*u*_l_ = 0.54 m s^–1^).

Having established how the level of detail at the channel scale influences the input for the electrolyser scale (in the form of χ_het_, χ_hom_, *FE*_C_2_H_4__, and *V*_Cell_ for a given *i*_tot_, *u*_g_, and *i*_hom_ (M2) or *u*_l_ (M3)), the propagation of the level of detail to the process scale is shown in terms of the *relative NPV* in Figure 4. The *relative NPV* (eq 33) compares the *NPV* (eq 27) for each model to the maximum *NPV* reached with model M1 (*NPV* ≈ 24 M$ at *i*_tot_ ≈ 600 mA cm^–2^). The calculations for the maximum *NPV* of model M1 can be found in section S6.1 with a discussion on the negative *NPV* in section S8. Therefore, a decrease or increase of the *NPV*_rel,Mx_ indicates the same relative change in the *NPV*, and hence, both terms will be used interchangeably in the following. In Figure 4 (a and b), the green lines depict the *NPV*_rel,M1_ calculated with the *no* channel model (M1), with the current density being the only variable input parameter to the electrolyser scale (see Table 2 and Figure 1). As described in previous literature,^6^ the current density in M1 solely influences the electrolyser area (eq 23) and therefore the investment costs leading to the expected trend of a steady increase in the *NPV* with an increase in current density as shown in Figure 4 a), eventually reaching the asymptotic value of 1. Contrary to model M1 an increase in current density leads to a decrease in the *NPV* for high current densities (see section S6.2), for both mechanistic models (M2 and M3) resulting in a clear optimum within the range of variation of the current density. A similar trend is observed when fixing the current density while varying the single channel gas flow rate, as shown in Figure 4b. While the single channel gas flow rate shows no effect on the *NPV* in model M1 as the input variables are fixed, both mechanistic models display again a clear optimum. The significance of the single channel gas flow rate on the *NPV* was also observed in the sensitivity analysis in section S2.6. This analysis reveals that the interdependencies of the input variables translate to important trade-offs on the process scale, which cannot be captured with fixed variable models such as model M1.

The maxima in the *NPV* for channel models M2 and M3 are explained through the interdependence of mass transfer limitation, heterogeneous conversion, and homogeneous consumption. Figure 4c shows this trade-off schematically in terms of investment and operating costs linked to the current density and single channel gas flow rate. From the previous section, we learned that low current densities retain low heterogeneous conversion rates and high selectivities. Further the high CO_2_ concentrations in the catalyst layer cause the mass transfer related overpotential (eq 13), and hence the overall cell potential (eq 22), to decrease. A low cell potential reduces the required power input, translating to lower operating costs for the electrolyser. However, due to the low conversion, larger electrode areas and separation units are required to maintain a specific throughput, which increases the investment costs. High current densities on the other hand increase the heterogeneous conversion rate and therefore reduce investment costs but also lead to an unwanted expense of electrons through the increased reduction of water in the hydrogen evolution reaction (eq 8), therefore increasing the operating costs. The trade-off between investment and operating costs is similarly observed for varying single channel gas flow rates. High single channel gas flow rates reduce the residence time in the channel and consequently reduce the heterogeneous conversion rate leading to an increased daily gas throughput to achieve the target production rate of ethylene. This in turn requires a larger separation unit increasing the investment costs. Low single channel gas flow rates, on the other hand, increase the heterogeneous conversion, lowering the daily gas throughput. However, this leads to a depletion of CO_2_ along the channel, which resulted in an increase toward the hydrogen evolution reaction. This in turn increases the operating costs of the electrolyser as electricity is now lost toward the parasitic side reaction.

The insights on the trade-offs explain the difference in the impact of current density and gas flow rate on the costs. The single channel gas flow rate mainly influences the heterogeneous conversion rates through the residence time in the channel and consequently, the investment costs of the gas separation unit and the operating costs of the electrolyser. The current density, however, influences both the investment and operating costs of the electrolyser as well as for the separation unit.

Mapping the *relative NPV* for all three models (M1–M3) over the space of varying current densities and single channel gas flow rates allows us to compare how the optimal operating areas vary with the level of mechanistic detail in the model. Figure 4d–f show that for all models low current densities and high single channel gas flow rates are not optimal based on the high investment costs. However, only models M2 and M3 additionally show a higher loss region for high current densities and low single channel gas flow rates. The *no* channel model (M1) does not display this trade-off due to a fixed conversion rate (Table 2) leading to fixed operating costs. Therefore, a clear optimum for the operating conditions is found for the models considering the interdependencies between the performance variables (indicated by the dots in Figure 4e and f).

### Optimization Results

**Figure 5:**
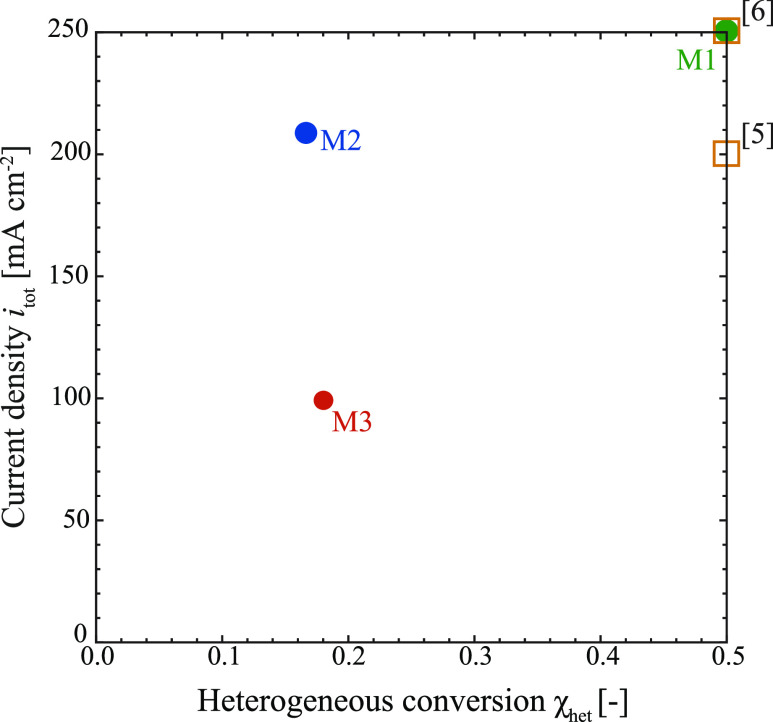
Optimal operation points for M1, M2, and M3 (circles) in terms of current density and heterogeneous conversion based on the optimal single channel gas flow rate as presented in Figure 4e and f. For M1, this point is found at the boundary of its domain (χ_het_ = 0.5). The target values based on literature^5,6^ are denoted as squares.

The pronounced impact of the current density and single channel gas flow rate on *NPV* has been discussed in the previous section. It was further shown that considering the interdependencies (model M2 and M3) between the performance variables leads to a trade-off between investment and operating costs which manifests in a clear optimum for the operating conditions. The optimization results are summarized in Table 3. Note that the optimal results for M1 are not shown as the conversion rate is fixed, and the optimal current density always lies at the upper constraint, i.e., at 250 mA cm^–2^. Figure 5 compares the optimization results with literature based operating targets. It can be seen that the optimal current density for M2 lies close to the values suggested as minimal-threshold in nonmechanistic techno-economic analysis (above 200 mA cm^–2^), while this value is considerably lower when modeling the consumption of CO_2_ as a function of the process conditions (M3). Here, the optimal current density lies at ≈100 mA cm^–2^, roughly half the value that is found for M2 and lower than the threshold values proposed in the literature.^5,6,13^ This is driven mainly by the prediction of a strong increase in unwanted homogeneous consumption at high current densities, rendering operation at lower current densities desirable. The observed product distribution for the formation of higher hydrocarbons and the reported increase in selectivity with increasing current density are neglected in this work. As the kinetic expressions (eqs 11–14) and the assumptions regarding the separation unit are the same in models M2 and M3, the trade-off for the heterogeneous conversion is the same in both models. Hence, the optimizer converges to a single channel gas flow rate, which facilitates a similar conversion rate for both models of slightly below 20%. This comparably low conversion is subject to how the kinetics are formulated in this work, leading to a strictly inverse relationship between the conversion and faradaic efficiency. Besides the simplification of the kinetics on the findings, other important assumptions are taken. In the remainder of the paper, we discuss their foreseen impact on our findings.

**Table 3:** Overview of the Optima for Models M2 and M3 As Shown in Figure 4e and f

Variable	Unit	M2	M3^a^
Input			
*i*_tot_	[mA cm^–2^]	209	99.2
*u*_g_	[m s^–1^]	0.05	0.02
Channel scale			
*V*_cell_	[V]	3.80	3.47
*FE*_C_2_H_4__	[-]	0.89	0.85
χ_het_	[-]	0.17	0.18
χ_hom_	[-]	0.04	0.02
Electrolyser scale			
*V̇*_r_	[m^3^ s^–1^]	1.23	1.14
*A*_r_	[m^2^]	2.57 × 10^3^	5.67 × 10^3^
*P*_r_	[MW]	20.4	19.5
*ṁ*_CO_2__	[kg yr^–1^]	1.40 × 10^3^	1.25 × 10^3^
Process scale			
*NPV*	[M$]	–22.0	–24.0

a For model M3, the obtained optimal values show a dependency on the initial guess with a deviation of less than 5%.

### Limitations of Results

The presented channel scale model is more advanced than the majority of CO2R models reported previously, yet several phenomena are not included such as liquid product formation and their migration to and oxidation at the anode, migration of bicarbonate/carbonate ions, and hence degassing of CO_2_ at the anode side. While we provide a detailed discussion on the influence of these phenomena on our findings in section S9, we highlight here the influence of migration and degassing of CO_2_. In anion exchange membrane electrolysers for example a significant amount of CO_2_ crosses over to the anode side in form of carbonate ions as the main charge carrying species under steady-state conditions.^32^ This increases the consumption rate of CO_2_ compared to the predictions of model M3,^34^ leading to lower optimal CO_2_ conversions. In bipolar membrane electrolysers this effect can be minimized at the cost of increasing the ohmic resistance and hence required cell potential,^33^ decreasing the overall *NPV* (see section S8). Further, we assume the investment and operating costs of the separation unit (PSA) to depend only on the overall required single channel gas flow rate and not on the composition of the gas stream itself. Additionally, the liquid products and potential cleaning steps of the electrolytes are not considered. Conceivably, including detailed models of the required post treatment units leads to higher investment costs and optimal CO_2_ conversion rates.

Considering the various assumptions underlying these results, the authors stress that the herein reported optimal values shall not be understood as newly proposed target values for the performance variables. Rather, they shall showcase how combining mechanistic and techno-economic models allows for design optimizations in the field of CO_2_ electrolysis. More importantly, they show how the level of mechanistic detail in such models strongly influences the resulting recommendations. In this sense, the distance between the optimum points for the models M1, M2, and M3 in Figure 5 can be understood as resulting from different levels of mechanistic understanding, while the distance between the optimum points and the literature recommendations results from a discrepancy between required and currently possible electrolyser performances.

## Conclusion and Outlook

Target values for performance variables for the low-temperature electrochemical conversion of CO_2_ have so far been derived from techno-economic analysis based on the assumption that the performance variables such as current density and faradaic efficiency are independent. In this work, we present a multiscale framework that incorporates mechanistic models of a GDE-based CO_2_ electrolyser to capture the interdependence between the performance variables required as input to the electrolyser scale: heterogeneous conversion, homogeneous consumption, faradaic efficiency, and cell voltage. This framework is used to perform a techno-economic assessment and optimization for a CO_2_-electrolysis-based process, revealing optimal target values for the performance variables that can strongly deviate from previously reported targets. For the herein chosen electrolyser design this manifests in an optimal current density of around half of commonly reported values. While it should be noted that the optima in this work are derived based on simplified reaction mechanisms and design considerations and therefore should not be taken as fixed optimum values for future electrolyser designs, the used approach nonetheless highlights the dependency of the mechanistic detail and interdependencies between performance variables on the economic viability of an electrolyser design. This work further presents a tool to evaluate electrolyser design choices based on an economic objective, which in its generic form can be applied to various electrolyser designs^18,44−47^ and CO_2_-reduction products, such as CO or formate.^19,25,48^ For different electrolyser designs and products, the modeling assumptions, and hence, the economic predictions, are highly dependent on the available data; therefore, it is important to (a) move toward more holistic, multiscale modeling approaches in the field of CO_2_ electrolysis and to (b) communicate measured or targeted electrolyser performance with all applicable boundary conditions, including achieved conversions.

## Data Availability

The full multiscale model is available via https://github.com/IsabellBagemihl/Multi-scaleModelElectrochemicalCO2Reduction.git.

## Supporting Information Available

Detailed description including the derivation and validation of the single channel model, all material and operating parameters, derivation of electrolyser scale model, sensitivity analysis toward technical variables and economic parameters, and discussion of selected parameters and assumptions and their effect on the findings (PDF)
