# Supplementary material for: Techno-economic Assessment of CO2 Electrolysis: How Interdependencies between Model Variables Propagate Across Different Modeling Scales
Source: ACS Sustain Chem Eng. 2023 Jun 28;11(27):10130–41. doi: 10.1021/acssuschemeng.3c02226 (PMC10337259; doi:10.1021/acssuschemeng.3c02226)
Supplement: Supplementary file 1 — sc3c02226_si_001.pdf [file sc3c02226_si_001.pdf]

Supporting Information:

Techno-economic assessment of CO_2 electrolysis: How interdependencies between model variables propagate across different modelling scales.

Isabell Bagemihl,[*,†,¶] Lucas Cammann,[†,¶] Mar Pérez-Fortes,[‡] Volkert van Steijn,[†] and J. Ruud van Ommen[†]

†*Department of Chemical Engineering, Delft University of Technology, Van der Maasweg 9, 2629 HZ Delft, The Netherlands*

‡*Department of Engineering Systems and Services, Delft University of Technology, Jaffalaan 5, 2628 BX Delft, The Netherlands*

¶*Contributed equally to this work*

E-mail: i.bagemihl@tudelft.nl

Total - 32 pages, 10 figures, and 6 tables

Figure S1-S10, Table S1-S6

Table S1: List of symbols.

Symbol	Description	Unit
Roman		
A_{r}	Required electrolyser area	m^2
$c^{ref}_{\mathrm{CO_2}}(aq)$	Reference concentration	$\mathrm{mol\,m^{-3}}$
c_{k}	Concentration of species k	$\mathrm{mol\,m^{-3}}$
C_{m}	Annual maintenance costs	$\$\ \mathrm{yr^{-1}}$
C_{op}	Annual operational costs	$\$\ \mathrm{yr^{-1}}$
C_{rev}	Annual revenue	$\$\ \mathrm{yr^{-1}}$
CF	Cash flow	$\$\ \mathrm{yr^{-1}}$
D_{k}	Diffusion coefficient of species k	$\mathrm{m^2\,s^{-1}}$
E^0_{r}	Standard potential of the reaction r	V
E_{r}	Applied potential vs RHE of the reaction r	V
$\dot{F}_{\mathrm{C_2H_4,target}}$	Molar flow rate of targeted C_2H_4 production	$\mathrm{mol\,s^{-1}}$
F	Faraday constant	$\mathrm{A\,s\,mol^{-1}}$
$FE_{\mathrm{C_2H_4}}$	Faradaic efficiency towards ethylene	-
H	Channel height	m
H_{c}	Catalyst layer thickness	m
H_{m}	Membrane layer thickness	m
$H_{\mathrm{CO_2,elec.}}$	Henry constant CO_2	-
i_{hom}	Additional current density for homogeneous consumption	$\mathrm{A\,m^{-2}}$
$i_{\mathrm{0,k}}$	Exchange current density of species k	$\mathrm{A\,m^{-2}}$
i_{k}	Partial current density of species k	$\mathrm{A\,m^{-2}}$
i_{tot}	Current density (sum of partial current densities)	$\mathrm{A\,m^{-2}}$
IR	Interest rate	-
k_{f}	Forward reaction rate constant	$\mathrm{m^3\,mol^{-1}\,s^{-1}}$
k_{r}	Reverse reaction rate constant	$\mathrm{s^{-1}}$

L	Channel length	m
M_{k}	Molar mass of species k	$\mathrm{kg\,mol^{-1}}$
$\dot{m}_{\mathrm{k}}$	Annual mass flow rate of species k	$\mathrm{kg\ yr^{-1}}$
$\dot{n}_{\mathrm{k,gl}}$	Molar flux across the gas-catalyst interface	$\mathrm{mol\,m^{-2}\,s^{-1}}$
$\dot{N}_{\mathrm{k,diff}}$	Diffusive species transport	$\mathrm{mol\,m^{-3}\,s^{-1}}$
$\dot{N}_{\mathrm{k,het}}$	Electrochemical production rate	$\mathrm{mol\,m^{-3}\,s^{-1}}$
$\dot{N}_{\mathrm{k,hom}}$	Homogeneous consumption rate	$\mathrm{mol\,m^{-3}\,s^{-1}}$
NPV	Net present value	\$
P	Pressure	Pa
P_{r}	Overall power consumption	W
R	Universal gas constant	$\mathrm{J\,mol^{-1}\,K^{-1}}$
t	Time	s
T	Temperature	K
TCI	Total capital investment	\$
u_{g}	Gas velocity	$\mathrm{m\,s^{-1}}$
u_{l}	Liquid velocity	$\mathrm{m\,s^{-1}}$
u_x	Flow velocity in x direction	$\mathrm{m\,s^{-1}}$
V_{cell}	Electrolyser cell potential	V
$\dot{V}_{\mathrm{r}}$	Volumetric flow rate through electrolyser	$\mathrm{m^3\,s^{-1}}$
W	Channel width	m
x	Coordinate in flow direction	m
y	Coordinate perpendicular to the flow direction	m
z_{r}	Amount of transferred electrons in reaction r	-

Greek		
α_r	Transfer coefficient of the reaction r	-
β	Fitting factor for the cost correlation of the PSA unit	-
$\delta_\mathrm{c}(x)$	Liquid boundary layer thickness	m
ϵ	Porosity	-
η_r	Overpotential of the reaction r	V
η_Ω	Ohmic losses	V
κ	Conductivity	$\mathrm{S\,m^{-1}}$
$\nu_{k,r}$	Stochiometric coefficient of species k in reaction r	-
τ	Tortuosity	-
χ_het	Heterogeneous conversion	-
χ_hom	Homogeneous consumption	-
χ_tot	Overall conversion	-

1 Homogeneous conversion in *simplistic* channel scale model M2

The *simplistic* channel scale model M2 is represented by a plug flow reactor in which the CO_2 flow is reacting at the channel wall in the electrochemical reaction (Eq. 9, main manuscript). The loss of CO_2 in the homogeneous carbonate reaction (Eqs. 15 - 16, main manuscript) is not explicitly modelled but simplified by introducing a fixed additional current density at the wall (i_{hom}) which leads to the following species balance for CO_2 based on Eq. 1 -2 (main manuscript)

$$\frac{\partial c_{\mathrm{CO_2,M2}}}{\partial x} = -\frac{2\left(i_{\mathrm{C_2H_4}} + i_{\mathrm{hom}}\right)}{12 F u_{\mathrm{g}} H} \tag{S1}$$

This approach allows to include a homogeneous consumption term, which solely depends on the gaseous flow rate for a fixed additional current density ($i_{\mathrm{hom}} = 50\,\mathrm{mA\,cm^{-2}}$).[S1,S2] To calculate the homogeneous consumption we need to know how much CO_2 is consumed by this additional current density. We determine this by solely considering the reaction due to the additional current density i_{hom} in Eq. S1. The change in CO_2 concentration over the channel length based on the additional current density is given by

$$\int_{c_{\mathrm{CO_2,hom,M2}}(x=0)}^{c_{\mathrm{CO_2,hom,M2}}(L)} dc = -\frac{2 i_{\mathrm{hom}}}{12 F u_{\mathrm{g}} H} \int_0^L dx. \tag{S2}$$

The homogeneous consumption for this simplification equals the total conversion (Eq. 18, main manuscript)

$$\chi_{\mathrm{hom}} = \frac{c_{\mathrm{CO_2,hom,M2}}(x=0) - c_{\mathrm{CO_2,hom,M2}}(x=L)}{c_{\mathrm{CO_2,hom,M2}}(x=0)} = \frac{2 i_{\mathrm{hom}} L}{12 F u_{\mathrm{g}} H} \frac{RT}{P}, \tag{S3}$$

with the concentration of CO_2 at the channel inlet given by the ideal gas law. For a single channel gas flow rate of 10 sccm and the herein used channel geometry the homogeneous consumption comes out to be $\chi_{\mathrm{hom}} = 0.13$ (Table 2, main manuscript).

2 Model parameters

The parameters for the simulations are summarised in Table S2.

Table S2: Input parameters for channel scale models.

Parameter	Description	Value	Unit	Reference
Operating conditions				
u_g	Gas velocity	$1.00 \cdot 10^{-3}$	$[m\,s^{-1}]$	-
u_l	Liquid velocity	0.54	$[m\,s^{-1}]$	-
η_c	Cath. overpotential	-0.70 to -0.90	[V]	S3
T	Temperature	300	[K]	-
P	Pressure	$1.00 \cdot 10^{5}$	[Pa]	-
Geometry				
ϵ	Porosity	0.70	[-]	S3
H	Channel height	$1.00 \cdot 10^{-3}$	[m]	S4
W	Channel width	0.01	[m]	S4
L	Channel length	0.10	[m]	-
H_c	Cat. layer thickness	$3.00 \cdot 10^{-6}$	[m]	S3
H_m	Mem. layer thickness	$115 \cdot 10^{-6}$	[m]	S5
Species/material properties				
D_{CO_2}	Diffusion coeff. CO_2	$1.91 \cdot 10^{-9}$	$[m^2\,s^{-1}]$	S6
D_{OH^-}	Diffusion coeff. OH^-	$5.30 \cdot 10^{-9}$	$[m^2\,s^{-1}]$	S6
$D_{CO_3^{2-}}$	Diffusion coeff. CO_3^{2-}	$0.92 \cdot 10^{-9}$	$[m^2\,s^{-1}]$	S6
$D_{HCO_3^-}$	Diffusion coeff. HCO_3^-	$1.91 \cdot 10^{-9}$	$[m^2\,s^{-1}]$	S6
$H_{CO_2,elec.}$	Henry constant CO_2	0.85 [a]	[-]	S7
Electrochemical properties				
κ_e	Electrolyte conduct.	5.50	$[S\,m^{-1}]$	S8
κ_m	Membrane conduct.	9.30	$[S\,m^{-1}]$	S5
$i_{0,OER}$	Exch. current density OER	$1.00 \cdot 10^{-7}$	$[A\,m^{-2}]$	S9
$c^{ref}_{CO_2}(aq)$	Reference concentration	34.0	$[mol\,m^{-3}]$	-
E^0_c	Standard eq. potential cathode	0.08	[V]	-
E^0_a	Standard eq. potential anode	1.23	[V]	-

[a] Converted to concentration form at standard conditions.

3 *Full* channel scale model M3

The electrochemical reactor setup under consideration is the gas diffusion electrode (GDE) cell with an electrolyte gap, as described in Figure S1. The channels of the GDE-cell are situated on a flowplate, where the gas-flow is directed co-currently to the electrolyte flow in the liquid flow chamber. The gas- and liquid flow chamber are separated by the gas-diffusion layer and the catalyst layer, whereas the liquid flow chamber itself is separated by a membrane. The *full* channel model M3 formulated in this work only encapsulates the domains which are considered dominant for the transport and reaction of the main reactants and products, those being the gas flow chamber, the catalyst layer, and the liquid boundary layer. Hence it is assumed that the height of the gas diffusion layer can be neglected for gas phase transport and that gaseous species do not diffuse into the bulk electrolyte. Other physical effects necessary to describe the overall cell performance are derived from approximate relationships. The domains in which this is done are shown in Figure S1 bordered by dashed red lines, while the compartments which are explicitly modeled are framed by solid red lines.

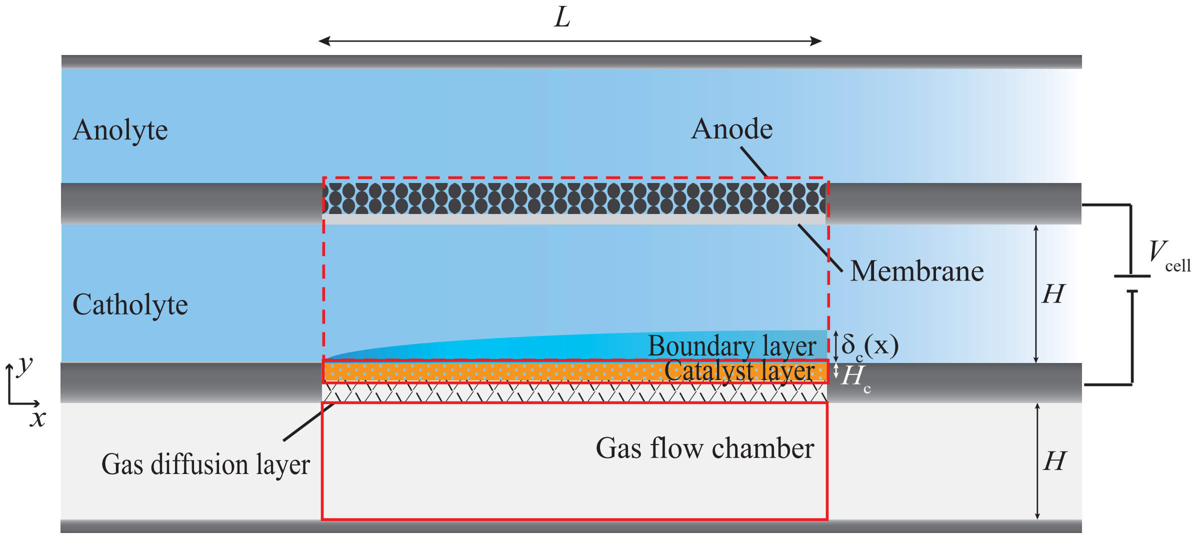

Figure S1: Schematic of a single channel in the considered GDE cell. Model domains which are treated explicitly have a solid red border, those for which approximate relationships are used have a dashed red border.

3.1 Derivation of governing gas channel equation

In this Section, the derivation of the governing equation for the gas channel (Eq. 1, main manuscript) is presented, starting from the generic form of a species balance and simplifying it by assuming unidirectional flow with convective transport dominating diffusive transport in the x-direction,

$$\frac{\partial c_\mathrm{k}}{\partial t} = -\frac{\partial c_\mathrm{k}}{\partial x} u_x(y) + D_\mathrm{k} \frac{\partial^2 c_\mathrm{k}}{\partial y^2}. \tag{S4}$$

Assuming steady state, and integrating over the channel height (y-direction), hereby exploiting the mean value theorem for integrals, we obtain the following expression

$$\begin{aligned} 0 &= -\frac{1}{H}\int_0^{-H} \frac{\partial c_\mathrm{k}}{\partial x} u_x(y)\, dy + \frac{D_\mathrm{k}}{H}\int_0^{-H} \frac{\partial^2 c_\mathrm{k}}{\partial y^2}\, dy, \\ \rightarrow 0 &= -\frac{\partial c_\mathrm{k}}{\partial x} u_\mathrm{g} + \frac{D_\mathrm{k}}{H}\left[\frac{\partial c_\mathrm{k}}{\partial y}\right]_0^{-H}. \end{aligned} \tag{S5}$$

Note that in Eq. S5, the definition of the *mixing cup velocity*[S10] has been used, denoted as u_g. It is equal to the gas velocity when assuming plug flow behaviour. The integral appearing in the second term can be readily evaluated by realising that at the gas-catalyst interface ($y = 0$) the equality of fluxes is imposed, while at the opposing wall ($y = -H$) a no-flux condition holds, i.e.

$$\begin{aligned} -D_\mathrm{k} \left.\frac{\partial c_\mathrm{k}}{\partial y}\right|_{x,y=0} &= \dot{n}_\mathrm{k,gl}(x), \\ \left.\frac{\partial c_\mathrm{k}}{\partial y}\right|_{x,y=-H} &= 0. \end{aligned} \tag{S6}$$

Inserting these boundary conditions into Eq. S5 yields

$$0 = -\frac{\partial c_\mathrm{k}}{\partial x} u_\mathrm{g} + \frac{D_\mathrm{k}}{H}\left(-\frac{\dot{n}_\mathrm{k,gl}(x)}{D_\mathrm{k}} - 0\right). \tag{S7}$$

Three species are considered to be present in the gas phase, those being C_2H_4, CO_2, and H_2. Therefore, three initial conditions are required at the channel inlet to solve all concentration profiles. These are given as:

$$c_{\mathrm{CO_2}}(x=0) = \frac{P}{RT}\,, \quad c_{\mathrm{H_2}}(x=0) = 0\,, \quad c_{\mathrm{C_2H_4}}(x=0) = 0. \tag{S8}$$

It is hence assumed that CO_2 is present as a pure ideal gas at the channel inlet.

3.2 Boundary conditions

The governing equations for the gas flow channel, the catalyst layer, and the boundary layer (Eqs. 1 - 6, main manuscript) give rise to a set of differential equations that are second order in y, each requiring two boundary conditions for a unique solution. These boundary conditions have to be met at the catalyst-gas interface at $y = 0$ and at the boundary layer/electrolyte interface at $y = H_\mathrm{c} + \delta_\mathrm{c}(x)$ (Figure S1), making it a boundary value problem. The physical behaviour of the considered species at these boundaries is mainly governed by the aggregate state of the respective species, hence it is sensible to group the boundary conditions accordingly. Since it is assumed that the gaseous products are instantly transported into the gas channel upon formation, only CO_2 needs to be accounted for. For the CO_2 solvated within the catalyst layer, thermodynamic equilibrium with the gas-phase is assumed at the catalyst-gas interface (computed via Henry's law), while no crossover is assumed at the extent of the boundary layer (no-flux condition). It is known that crossover of CO_2 into the electrolyte may occur, however this is neglected in this case. Hence, the boundary conditions for CO_2 can be written as

$$\begin{aligned} c_{\mathrm{CO_2}}(x, y=0) &= c_{\mathrm{CO_2},g}(x) H_{\mathrm{CO_2},elec.}\,, \\ \left.\frac{\partial c_{\mathrm{CO_2}}}{\partial y}\right|_{x, y=H_\mathrm{c}+\delta_\mathrm{c}(x)} &= 0 \end{aligned} \tag{S9}$$

The Henry constant $H_{CO_2,elec.}$ is hereby approximated to be that of CO_2 in pure water at ambient conditions. For ionic species, the boundary conditions are opposed to those of the gaseous species. Ionic species cannot diffuse from the catalyst into the gas-phase, hence a no flux condition is imposed on the catalyst-gas channel interface. The concentration of ionic species at the extent of the boundary layer is set to be the equilibrium concentration in the bulk electrolyte, which depends on the concentration of the electrolyte. The boundary conditions for ionic species follow as

$$\begin{aligned} \left.\frac{\partial c_k}{\partial y}\right|_{x,y=0} &= 0, \\ c_k(x, y = H_\mathrm{c} + \delta_\mathrm{c}(x)) &= c_{k,elec.}. \end{aligned} \tag{S10}$$

Figure S2 qualitatively depicts the concentration profiles of CO_2 and an exemplary ionic species in the catalyst and boundary layer together with the corresponding boundary conditions at two different x-positions along the channel. Note how the boundary layer is further extended at the second position and how the gas phase concentration of CO_2 is further depleted at that point.

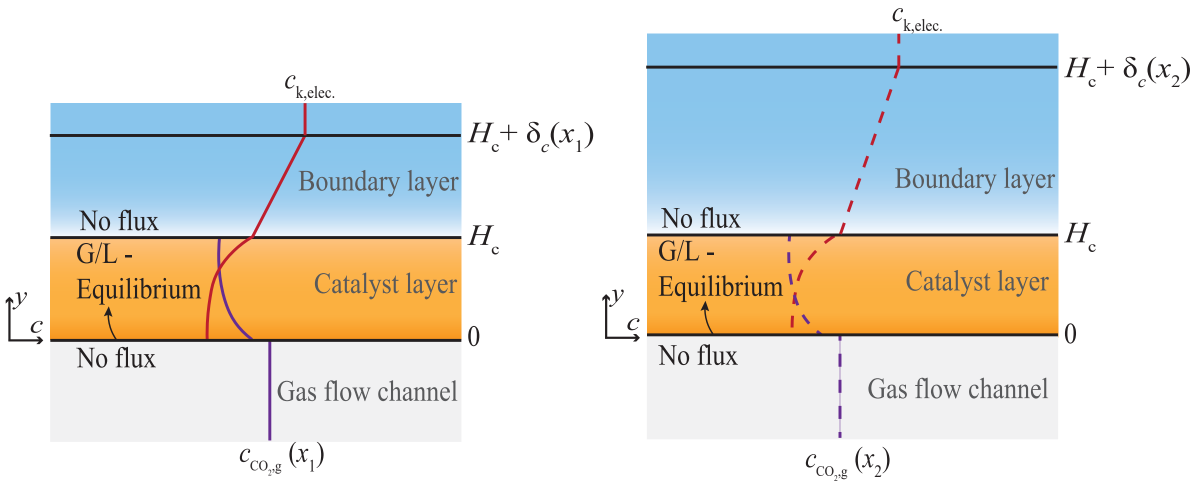

Figure S2: Qualitative depiction of the boundary conditions at two different channel positions, x_1 and x_2, with x_2 being further downstream. Axis are not at scale.

3.3 Kinetic parameters

Heterogeneous reactions

Given that C_2H_4 and H_2 are considered as the only products (i.e. not including the whole range of carbonaceous products which can form on a copper catalyst) it is refrained from estimating the heterogeneous reactions parameters for every possible reaction, and instead, an overall current-voltage relationship for the cathode side of the cell is used. The reason for this simplification is twofold:

1. The local reaction environment is known to strongly influence product distribution. To the best of the author's knowledge, there exists no kinetic framework which can account for this.
2. A simplistic kinetic approach is deemed favourable for comparing different operational modes for large scale CO_2 electrolysers, as intended in this work.

To obtain the desired estimate of a possible *i*-*E* curve for the single channel models M2 and M3, experimental data reported by Tan et al.[S3] is fitted through the Butler-Volmer equation

$$i_{\mathrm{tot}} = i_0 \exp\left(-\frac{\alpha_{\mathrm{c}} F}{RT}\left(E_{\mathrm{c}} - E_{\mathrm{c}}^0\right)\right) \tag{S11}$$

using a least squares minimisation with α_{c} and i_0 as fitting parameters. The experiments were conducted with a GDE-based flow electrolyser with copper nanoparticles on carbon paper and 1M $KHCO_3$ as catholyte.[S3] This study has been chosen as the experimental setup is well defined and representative of the modelling domain considered herein. The obtained values of the fitting parameters and their 95% interval are presented in Table S3. The resulting *i*-*E* curve together with the 95% confidence is plotted in Figure S3. Contrary to the experimental data ethylene is considered as the only carbonaceous product. The fitted kinetic constants shown in Table S3 are taken as the kinetic constants for calculating the total current density. Since CO_2 is mass transfer limited the partial current density for ethylene

is then given by the mass-transfer dependent Butler-Volmer equation which includes the concentration overpotential based on the Nernst equation (see Eq. 13, main manuscript) and shares the same Butler-Volmer constants as the total current density and the partial current density for the hydrogen formation. This simplifying assumption for the kinetic constants is motivated by the difficulty in fixing the individual kinetic constants for each cathode reaction in the Butler-Volmer equation as discussed by Brée et al.[S5]. The uncertainty in determining the kinetic constants arises from the data scarcity and that reported values vary drastically for different experimental studies, as the kinetic constants are difficult to decouple from the mass transport phenomena occurring in the cell.[S11] By simplifying the kinetic constants as explained above the partial current densities in this study can be directly calculated with the one set of fitted kinetic constants. The influence of this assumption is discussed in the following Section S3.4.

It can be seen that, while the experimental data points seem to be well represented with the Butler-Volmer equation with $\alpha_c = 0.26$ and $i_0 = 0.022\,\mathrm{mA\,cm^{-2}}$ as fit parameters, the confidence intervals $\alpha_{c,95}$ and $i_{0,95}$ are rather large. In fact, the 95 % confidence interval $\alpha_{c,95}$ exceeds the lower limit drops below 0. These large values for the confidence intervals of both fit parameters can be attributed to the small number of data points, a problem also encountered in similar CO_2RR modelling studies.[S5] While many kinetic studies of CO_2RR on copper electrodes have been conducted, the number of studies utilising GDE cells at high current densities is still limited and therefore, few studies are available containing more data points. Considering the restricted pool of reported experiments, the data provided by Tan et al.[S3] seem the best fit for the current purpose.

Table S3: Fitting parameters and their 95% confidence interval obtained by fitting the Butler-Volmer equation (Eq. S11) to the experimental $i - E$ curve by Tan et al.[S3].

Parameter	Unit	Value
α_{c}	[-]	0.26
$\alpha_{\mathrm{c},95}$	[-]	−0.28 to 0.79
i_0	[mA cm^{-2}]	0.022
$i_{0,95}$	[mA cm^{-2}]	−0.41 to 0.46

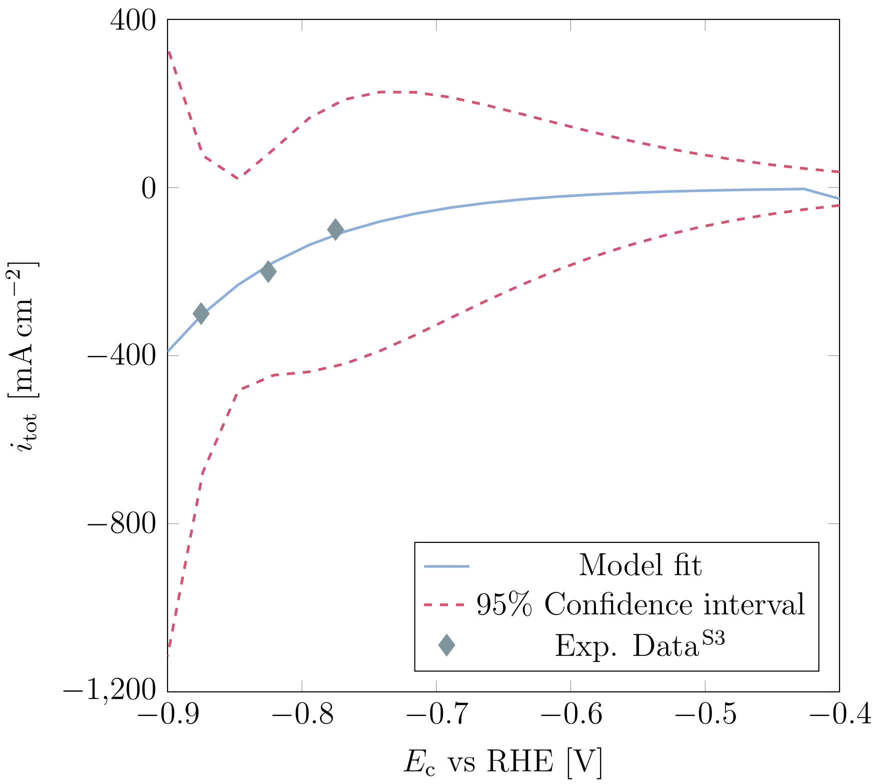

Figure S3: Experimental data[S3] and model fit together with 95% confidence interval.

Homogeneous reactions

The kinetic constants for the homogeneous buffer reaction are taken from Schulz et al.[S12] and are corrected for the electrolyte salinity.[S13] A summary of the values can be found in Table S4 for 1M $KHCO_3$ electrolyte solution.

Table S4: Reaction rate constants for homogeneous buffer reactions.

Forward rate [$m^3\,mol^{-1}\,s^{-1}$]	Reverse rate [s^{-1}]
$k_{f1} = 5.93$	$k_{r1} = 1.34 \cdot 10^{-4}$
$k_{f2} = 1.00 \cdot 10^{-5}$	$k_{r2} = 2.15 \cdot 10^{-4}$

3.4 Comparison of the *full* channel model M3 prediction with literature

The two modelling studies[S4,S14] on CO_2 electrolyser designs which similar to this work resolve the local effects along the channel length are not straightforwardly comparable to the herein presented results as they consider a different catalyst and therefore products with a different amount of electrons transferred per reaction. Yang et al.[S14] consider the formation of $HCOO^-$, while Kas et al.[S4] consider a silver catalyst with the main product being CO. Both of these reduction products require a 2 electron transfer reaction while the reaction chosen in this study towards ethylene requires a 12 electron transfer reaction. Further, the kinetics for the heterogeneous electrochemical reaction are simplified under the assumption that either CO_2 is reduced or H_2 is formed which is comparable to a constant current (galvanostatic) control (see Section "Heterogeneous reactions", main manuscript). Kas et al.[S4] presented for the first time the trade-off between heterogeneous conversion and homogeneous consumption of CO_2. As this trade-off is the main driver for the herein acquired results, their study was chosen as a point of comparison to validate the single channel model. For this purpose, the liquid flow rate and the channel length are adapted in this Section to the ones presented by Kas et al.[S4] to $1\,mL\,min^{-1}$ and 1 cm. Further, the model developed in this work is adjusted from a 12 electron transfer to 2 electron transfer reaction for the purpose of comparison in this Section. The predicted heterogeneous conversion and homogeneous consumption for different single channel gas flow rates are compared in Figure S4. Additionally, to compare the impact of the simplified reaction kinetics on the overall trends, the reaction kinetics in model M3 are changed to the Butler-Volmer kinetics and its constants, as presented in the

work by Kas et al.[S4] and referred to as BV kinetics in Figure S4.

The results of this work and the work by Kas et al.[S4] show a comparable trend for varying single channel gas flow rates, while the heterogeneous conversion for increasing current densities is lower in this work. As expected, the homogeneous consumption rate is then overpredicted. This is most likely rooted in the assumption of a fully flooded catalyst layer in this work compared to an ideally wetted catalyst layer in the work by Kas et al.[S4]. Weng et al.[S15] show that for a fully flooded catalyst layer the CO_2 concentration, and hence, faradaic efficiency, decrease for higher current densities compared to an ideally wetted catalyst layer due to an increase in local pH and the bicarbonate buffer reaction. It is therefore, no surprise that the homogeneous consumption due to the bicarbonate buffer reaction is predicted higher with our model compared to the work by Kas et al.[S4], leading similarly to a decreased heterogeneous conversion rate. Whether, the catalyst layer is fully flooded, partially flooded, or ideally wetted, depends on multiple parameters such as GDE fabrication, operating conditions, and duration of the reaction. Generally, a fully or partially flooded catalyst layer is expected under long term operation. Sisler et al.[S2] note that the loss of CO_2 due to the homogenous consumption is often underestimated. Therefore a fully flooded catalyst layer, which present the most restricted operating regime, was chosen for this work.

Comparing the predictions for conversion and consumption of this work for the incorporation of the full Butler-Volmer kinetics (dashed line) and the simplified Butler-Volmer kinetics (dotted line) in Figure S4, we observe that the conversion is predicted similar by both approaches while the consumption is overestimated in the case of the simplified kinetics. This is expected as for the full Butler-Volmer kinetics a fixed electrode potential is applied leading to a fixed partial current density for hydrogen and hence a fixed flux of OH^- along the electrode. The partial current density towards CO is changing along the electrode with the change in CO_2 concentration. This leads to a varying total current density for the full Butler-Volmer kinetics, which is averaged over the length for varying cathode potentials.

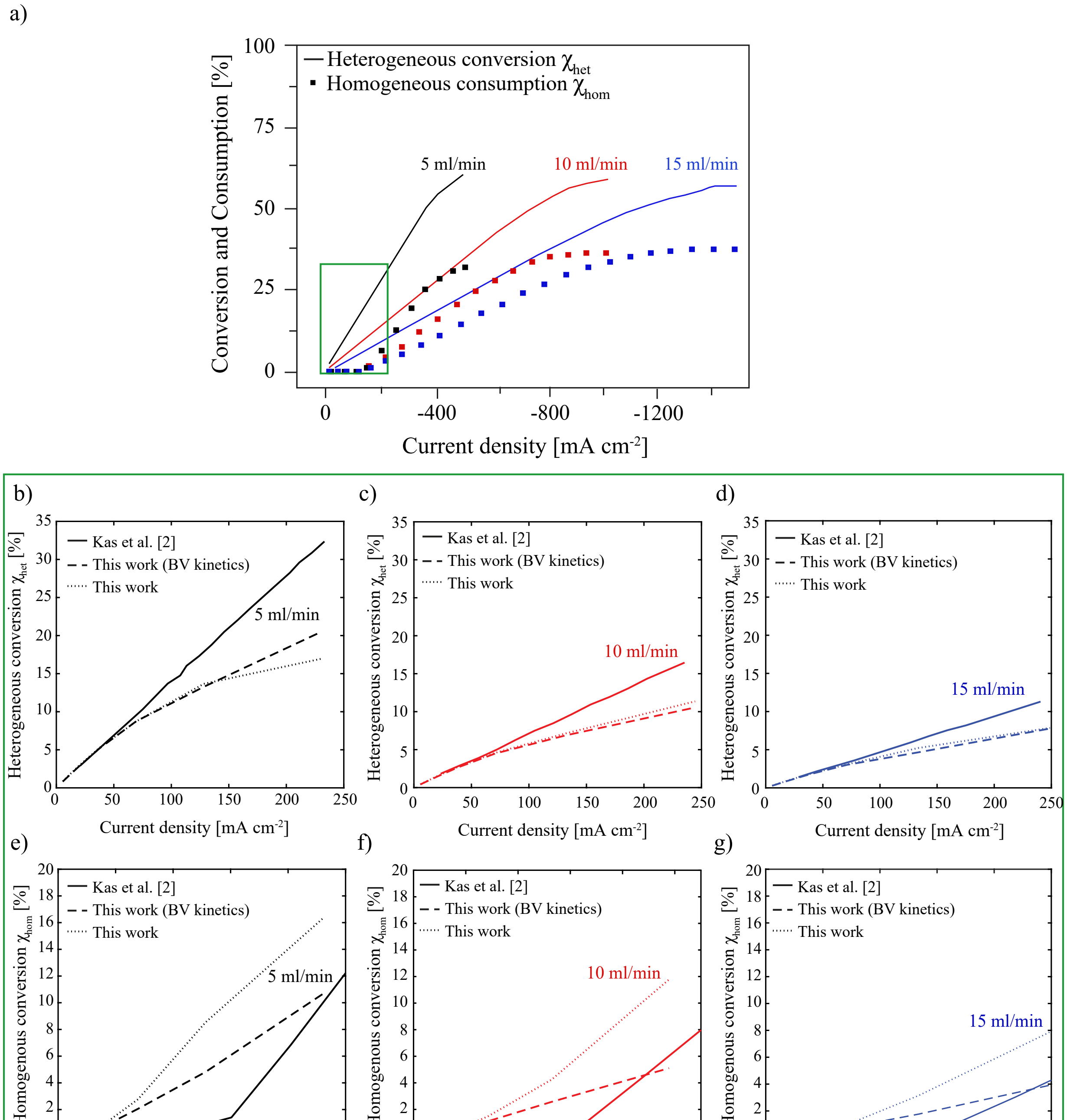

Figure S4: Numerical results for the heterogeneous electrochemical conversion from CO_2 to CO and the homogeneous consumption of CO_2 in the carbon equilibrium reaction from Kas et al.[S4] a). Zoomed in region for the relevant current density range used in this study comparing the numerical results from Kas et al.[S4] with the numerical results obtained with the *full* channel model M3 in this work for varying single channel gas flow rates b)-g). The numerical results for M3 considering the Butler-Volmer kinetics uses the values reported by Kas et al.[S4] (dashed lines), while the simplified kinetics (dotted line) are selected and used in this work.

The simplified Butler-Volmer kinetics similarly predict a varying partial current density of CO depending on the CO_2 concentration, leading to a similar prediction in heterogeneous conversion. Contrary, to the full Butler-Volmer kinetics, in the current paper, a fixed total current density is assumed over the electrode length. This assumption leads to an increase in hydrogen formation and production of OH^-. The increase in pH is believed to be the main reason for the observed increased homogeneous consumption rate. It is further interesting to note that even though this also affects the local CO_2 concentration, the impact on the heterogeneous conversion is negligible.

To further test the validity of the herein taken assumption and the simplification of the Butler-Volmer kinetics, the predicted partial current density towards ethylene with varying flow rates is compared to the experimental data by Tan et al.[S3] in Figure S5 a). In the work by Tan et al.[S3] a copper catalyst, which forms various C1 and C2+ products, is studied. In the current study ethylene is considered as the only CO_2 reduction product (which allows to simplify the post-process steps). We note that a catalyst which selectively produces ethylene has so far not been reported and hence presents a simplification in this study. For a point of

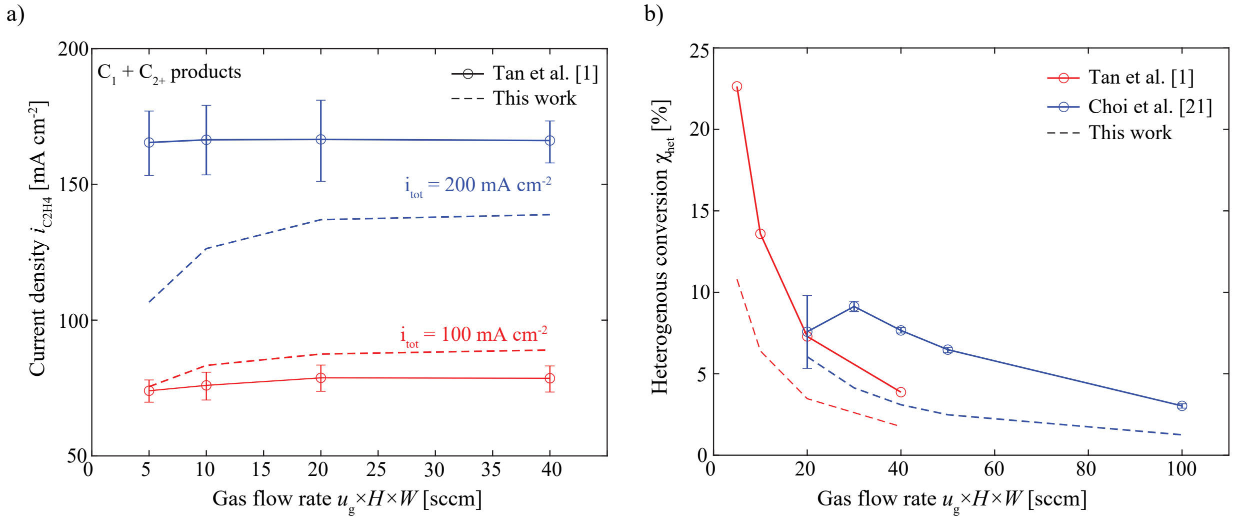

Figure S5: Numerical results for varying flow rates of a) the partial current density of ethylene in comparison to the sum of the partial current densities of C products obtained experimentally in the work by Tan et al.[S3], b) the heterogeneous conversion in comparison to the experimental data by Tan et al.[S3] and Choi et al.[S16].

comparison the sum of the partial current densities for the C1 and C2+ products from Tan et al.[S3] is taken and compared to the predicted current density towards ethylene. Figure S5 a) shows that for a total current density of $100\,\mathrm{mA\,cm^{-2}}$, the partial current density with varying flow rate is predicted well, while for a total current density of $200\,\mathrm{mA\,cm^{-2}}$, especially for low single channel gas flow rates, the partial current density towards ethylene is underpredicted. We note that the amount of electrons transferred varies widely for the individual C1 and C2+ products which could be a reason for the observed difference in partial current densities. Comparing the predicted heterogeneous conversion for varying single channel gas flow rates with the experimental data by Tan et al.[S3] and Choi et al.[S16] shows a similar trend while the absolute values are being under predicted. This has already been observed in Figure S4 for the comparison with the data from Kas et al.[S4] and the discrepancy in absolute values might similarly be due to the assumption of a fully flooded catalyst layer which might not be the case for the reported experimental values.

Conclusively, we note that while the absolute values cannot be predicted with the herein presented model and assumptions, the trends and trade-offs follow the experimentally and numerically observed trends. In this light, we reiterate that the current paper aims to show the trade-offs between conversion and consumption and their impact across scales and does not aim to provide new target values for electrolyser development as absolute values highly depend on the modelling assumptions, as shown above and throughout this study. Therefore, the sensitivity and accuracy of the proposed model is adequate to the purpose of the current paper.

4 Electrolyser scale model

The required electrolyser area, A_r is the most important variable for the capital investment costs and depends on the desired production rate according to

$$\dot{F}_{\mathrm{C_2H_4,target}} = A_\mathrm{r} \int_0^{H_\mathrm{c}} < \dot{N}_{\mathrm{C_2H_4,het}} > dy, \tag{S12}$$

with $< \dot{N}_{\mathrm{C_2H_4,het}} >$ the channel length-averaged rate. Solving the integral in Eq. S12 with Eq. 11 (main manuscript) leads to

$$\dot{F}_{\mathrm{C_2H_4,target}} = A_\mathrm{r} \frac{< i_{\mathrm{C_2H_4}} >}{12F}, \tag{S13}$$

with $< i_{\mathrm{C_2H_4}} >$ the channel length-averaged current density. The required electrolyser area A_r as presented in Eq. 23 of the main manuscript is obtained by combing Eq. S13 with the definition of the faradaic efficiency

$$FE_{\mathrm{C_2H_4}} = \frac{i_{\mathrm{C_2H_4}}}{i_{\mathrm{tot}}}. \tag{S14}$$

Assuming that the number of channels linearly scales with the reactor performance the relation between the required electrolyser area A_r and the volumetric gas flow rate $\dot{V}_\mathrm{r}$ leaving the electrolyser is given as

$$\dot{V}_\mathrm{r} = u_\mathrm{g} H \frac{A_\mathrm{r}}{L}. \tag{S15}$$

Knowing that $\dot{F}_{\mathrm{C_2H_4,target}} = c_{\mathrm{C_2H_4}}(x = L)\dot{V}_\mathrm{r}$ the faradaic efficiency can further be expressed in terms of the gas velocity and product concentration at the channel outlet (Eq. 21, main manuscript) by rearranging Eq. S14 with Eq. S15. The volumetric gas flow rate $\dot{V}_r$ (Eq. 24, main manuscript) leaving the electrolyser and entering the PSA unit can further be expressed

from the definition of the heterogeneous conversion (Eq. 19, main manuscript)

$$\chi_{\text{het}} = \frac{2c_{\text{C}_2\text{H}_4}(x=L)}{c_{\text{CO}_2}(x=0)}\frac{\dot{V}_\text{r}}{\dot{V}_\text{r}} = \frac{2\dot{F}_{\text{C}_2\text{H}_4,\text{target}}}{c_{\text{CO}_2}(x=0)\dot{V}_\text{r}}. \tag{S16}$$

The electrolyser unit is embedded in a Reaction-Separation-Recycle process (see Figure S6), in which the flowrate fed into the reactor is composed of an external stream ($\dot{m}_{\text{CO}_2,\text{in}}$) and a recycle stream ($\dot{m}_{\text{CO}_2,\text{recycle}}$). The magnitude of the recycle stream depends on the purity of the end product and the achieved conversion within the reactor itself. Perfect separation for the CO_2 stream in the PSA unit is assumed. For the current purpose, assuming a perfect separation is justifiable since this is *a)* commonly done for preliminary design optimisations[S17] and *b)* the separation efficiencies for PSA are reported to be quite high for similar feed streams.[S18] The required CO_2 supply $\dot{m}_{\text{CO}_2}$ then depends on the achieved conversion rate. Rewriting the conversion equations (Eq. 18 - 19, main manuscript) with $c_\text{i} = \dot{m}_\text{i}/(M_\text{i}\dot{V}_\text{r})$ in terms of mass flow rates under the assumption that the flow velocity is not changing from the electrolyser inlet to outlet (see Section S3) gives

$$\chi_{\text{het}} = 2\frac{\dot{m}_{\text{C}_2\text{H}_4,\text{target}}}{M_{\text{C}_2\text{H}_4}}\frac{M_{\text{CO}_2}}{\dot{m}_{\text{CO}_2} + \dot{m}_{\text{CO}_2,\text{recycle}}}, \tag{S17}$$

$$\chi_{\text{tot}} = \frac{\dot{m}_{\text{CO}_2}}{\dot{m}_{\text{CO}_2} + \dot{m}_{\text{CO}_2,\text{recycle}}}. \tag{S18}$$

Inserting Eq. S17 in Eq. S18 and rearranging yields the required supply of fresh CO_2 to the electrolyser

$$\dot{m}_{\text{CO}_2} = 2\dot{m}_{\text{C}_2\text{H}_4,\text{target}}\frac{\chi_{\text{tot}}}{\chi_{\text{het}}}\frac{M_{\text{CO}_2}}{M_{\text{C}_2\text{H}_4}}, \tag{S19}$$

which together with Eq. 20 (main manuscript) gives the annual consumption rate of CO_2 (Eq. 25, main manuscript).

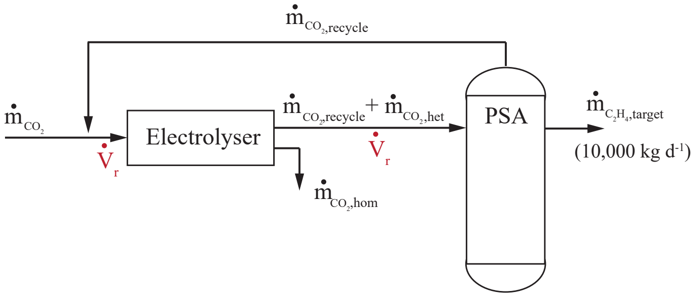

Figure S6: Conceptual flow configuration showing the CO_2 containing streams.

5 Additional constraints

Voltage losses Mitigating voltage losses is crucial for effective cell operation. These losses are constituted by required overpotentials to drive the reactions at the desired rate, as well as ohmic losses and make up the required cell voltage according to Eq. 22 (main manuscript). At the cathode side, concentration overpotential $\eta_{con,c}$ does not have to be taken into account due to the use of the concentration dependent Butler-Volmer equation (see Eq. 13, main manuscript). The activation overpotential is hence given as

$$\eta_{\mathrm{c}} = E_{\mathrm{c}} - E_{\mathrm{c}}^{0}, \tag{S20}$$

with the cathode potential E_{c} vs RHE (reversible hydrogen electrode). The cathode overpotential can be calculated for a given current density i_{tot} from the Butler-Volmer kinetics (Eq. 12, main manuscript).

For the OER occurring at the anode, modelling studies have reported negligible contributions of the concentration overpotential $\eta_{con,a}$, which is herein neglected.[S5,S19] The anodic activation overpotential on the other hand can become considerable, and must be taken into account. It can be approximated via the following expression

$$\eta_{\mathrm{a}} = \frac{RT}{0.5F} \sinh^{-1}\left(\frac{i_{\mathrm{tot}}}{2 i_{0,\mathrm{OER}}}\right), \tag{S21}$$

which is referred to as the *hyperbolic sine approximation.* This approximation is exact if the anodic and cathodic charge transfer coefficients in the Bulter-Volmer equation are equal.[S20] Note that the OER is the only reaction occurring at the anode, meaning that $i_{0,\mathrm{OER}}$ is equal to the cell current density. Ohmic losses are calculated via Ohm's law accounting for the conductivity of the membrane and the electrolyte

$$\eta_{\Omega} = i_{\mathrm{tot}}\left(\frac{H}{\kappa_{\mathrm{e}}} + \frac{H_{\mathrm{m}}}{\kappa_{\mathrm{m}}}\right), \tag{S22}$$

in which H_m is the membrane thickness, and κ_e, and κ_m are the conductivity of the electrolyte and membrane, respectively. Values can be found in Table S2.

6 Process scale model

6.1 Limiting NPV for model M1

As a comparative figure between models M1 to M3, the *relative NPV* (Eq. 33, main manuscript) is introduced, which uses $\max(NPV_\mathrm{M1})$ as a reference value. The NPV for each model is calculated based on the multi-scale model introduced in the main manuscript in the section "Multi-scale model". In Figure S7 the NPV for the *no channel* model M1 is plotted against the current density showing that the NPV reaches 99% of its final value at $\approx$ 600 $\mathrm{mA\,cm^{-2}}$ at $\approx$ -24 M$, which is taken as $\max(NPV_\mathrm{M1})$ in Eq. 33 in the main manuscript.

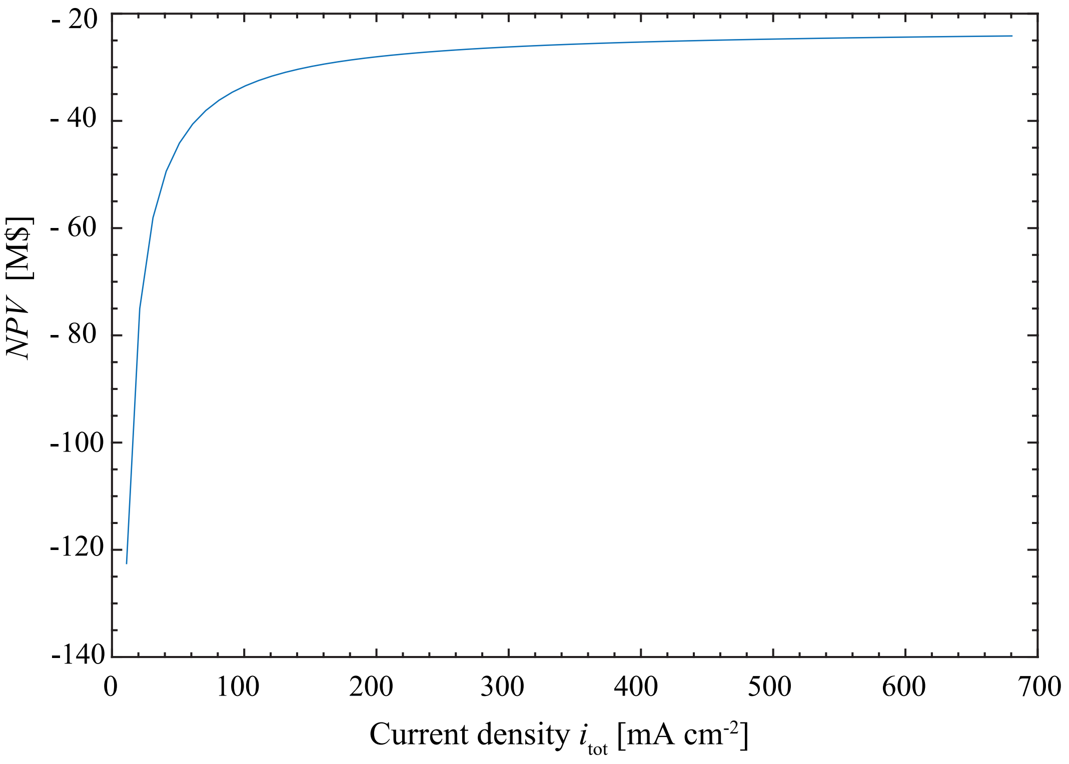

Figure S7: NPV for the *no channel* model M1 as a function of current density.

6.2 Sensitivity analysis

A sensitivity analysis is performed to evaluate the influence of selected process variables and economic parameters on the economic indicator, the NPV. These parameters and their ranges of variation are listed in Table S5. The results for the *simplistic* (M2) and *full* (M3) channel model are shown in Figure S8.

The NPV is negative for all (individual) ranges of variation. The flow rate and total current density show a significant influence on the obtained NPV, especially for model M3. The sensitivity analysis also shows a significant influence of the electricity costs on the NPV. This matches with conclusions from previous techno-economic studies, which conclude that low electricity prices are essential for the profitable production of higher hydrocarbons via electrochemical CO_2 reduction. The variation in selling price, CO_2 price, and production rate show a lower but also significant influence on the NPV.

Incorporating the channel scale model allows to study the influence of flow rate compared to other studies as this variable now influences the conversion and consumption rates, which are commonly fixed model inputs. The total current density influences the cell voltage, conversion, consumption and faradaic efficiency. An increase in total current density shows a negative effect on the NPV, while a decrease in total current density seems to improve the NPV. This observation is contrary to results reported in other techno-economic studies[S21–S23] in which an increase in current density generally increases the NPV. The influence of the current density and single channel gas flow rate on the NPV is further discussed in the "Results and Discussion" section of the main manuscript.

Table S5: Base case and variation ranges for sensitivity analysis.

Sensitivity variables	better	base	worse
Gas flow rate [sccm]	50	10	5
Total current density [$\mathrm{mA\,cm^{-2}}$]	300	200	100
Selling price ethylene [$ $\mathrm{kg^{-1}}$]	+15%	1.3	-15%
Electricity price [$ $\mathrm{kW^{-1}\,h}$]	0.02	0.03	0.04
CO_2 price [$ $\mathrm{kg^{-1}}$]	0	0.04	0.07
Electrolyser cost [$ $\mathrm{m^{-2}}$]	450	920	1840
PSA cost [$ $\mathrm{m^{-2}}$]	-20%	1990000	+20%
Interest rate [-]	+20%	0.1	-20%
Production rate [$\mathrm{kg\,d^{-1}}$]	-20%	10000	+20%
Electrolyser lifetime [yr]	-20%	20	+20%

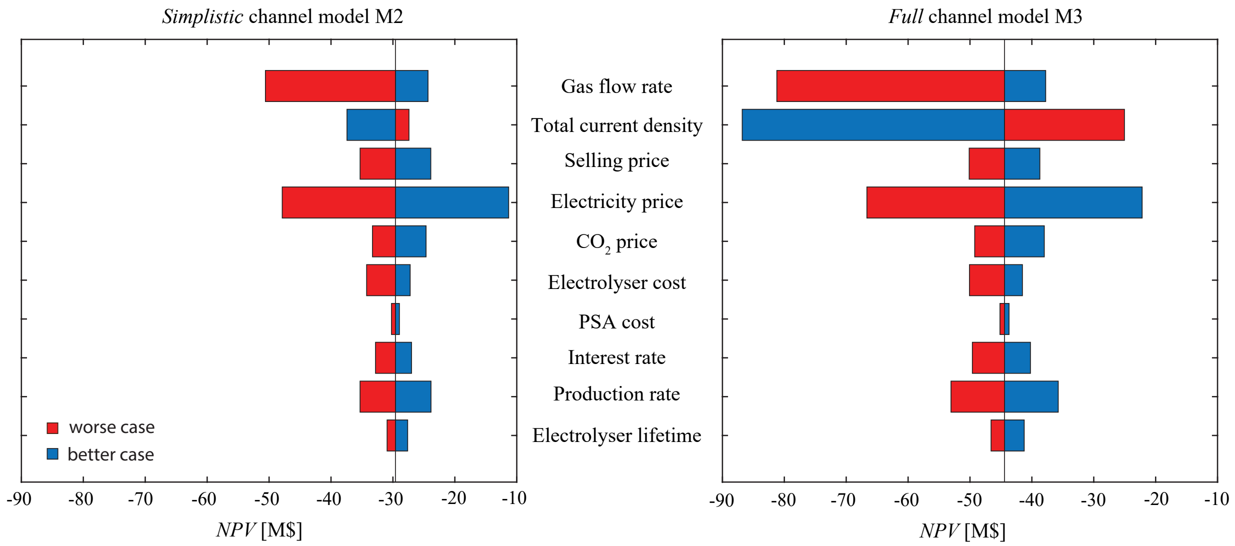

Figure S8: Sensitivity analysis of results for CO_2 to ethylene (Table S5).

7 Scaling relation between heterogeneous conversion and current density

The *simplistic* model M2 does not explicitly model the catalyst layer and describes the electrolyser solely as channel flow with a reacting wall. The reaction rate at the wall is described by the partial current density towards C_2H_4 (Eq. 13, main manuscript). Combining Eq. S1 (omitting the i_{hom} term) and Eq. 13 gives

$$\frac{\partial c_{\text{CO}_2}}{\partial x} = -\frac{i_{\text{tot}}}{6F} \frac{c_{\text{CO}_2}}{c^{ref}_{\text{CO}_2}(aq)} \frac{1}{u_{\text{g}} H}. \tag{S23}$$

Integrating over the reactor length x with the boundary condition $c_{\text{CO}_2}(x=0) = c^0_{\text{CO}_2}$ gives the expected exponential trend for the concentration of CO_2 over the reactor length

$$c_{\text{CO}_2}(x) = c^0_{\text{CO}_2} \exp\left(-\frac{i_{\text{tot}}}{6F c^{ref}_{\text{CO}_2}(aq)} \frac{x}{u_{\text{g}} H} \right). \tag{S24}$$

For low conversions, the CO_2 concentration drops almost linearly with the total current density i_{tot} for a fixed gas velocity u_{g}. With the heterogeneous conversion rate in turn depending linearly on the CO_2 concentration (Eq. 19, main manuscript), this explains the observed linear trend between heterogeneous conversion rate and total current density

8 Selection of fixed performance variables for model M1

Within this work, all NPVs for the electrochemical CO_2 reduction process to C_2H_4 are found to be negative. This stands in contrast to other publications, such as the seminal paper of Jouny et al.[S22]. The discrepancy can be explained upon inspection of the employed fixed performance variables for the electrolyser. While prior studies have attempted to find fixed performance variables which render the electrolysis process profitable, this study takes into account their interdependency. While the performance variables are not resolved through the channel scale model of the *no channel* model M1, the interdependencies are taken into account by choosing the fixed performance variables based on a combination of experimental results ($FE_{C_2H_4} = 0.70$)[S24] and calculations taken in this work for the cell potential ($V_{cell} = 3.69\,V$, Eq. 22 in the main manuscript), which is based on the threshold current density of $200\,mA\,cm^{-2}$ from Jouny et al.[S22]. Table S6 directly compares the selected performance targets used by Jouny et al.[S22] to the ones used in model M1 of this work. These performance targets act as an input to the electrolyser scale. The NPV is then calculated according to the Eqs. 18 - 27 in the main manuscript, similar to the *no channel* model M1. As stated, no positive NPV were obtained for the values used in this work as illustrated by the black line in Figure S9. To confirm that our electrolyser and process models also yield positive NPVs we computed the NPVs with model M1 for the targets used by Jouny et al.[S22] as listed in Table S6 (red line in Figure S9). To illustrate the importance of V_{cell}, we also computed the NPVs for the fixed cell voltage chosen in this work of $V_{cell} = 3.69\,V$ (green line in Figure S9) instead of the 2.00 V from Jouny et al.[S22]. Figure S9 shows that even with relatively optimistic values used by Jouny et al.[S22] for both the heterogeneous conversion and the faradaic efficiency, only the calculations with a cell voltage of 2.00 V achieve a positive NPV for a current density above $100\,mA\,cm^{-2}$. The NPV increases with increasing the current density, as the capital investment costs decrease with increasing current density for

a fixed cell potential. An increase in fixed cell potential reduces the overall NPV, while the trend with varying current density does not change. The cell potential drives the capital investment costs and is closely linked to the electricity price. When the cell potential is not fixed but its dependency on current density is considered (black line in Figure S9) a clear trade-off for increasing current densities is observed. The intersect at around 200 $mA\,cm^{-2}$, which equals a cell potential of $V_{cell} = 3.69\,V$ shows that an increasing cell voltage decreases the NPV. Conclusively, the choice of the fixed performance variables has a strong influence on the NPV of the electrolysis process, with the cell voltage being one of the main cost drivers. This is in line with the sensitivity results reported by Jouny et al.[S22].

Table S6: Comparison of performance targets used in prior work by Jouny et al.[S22] and in model M1 of this study.

	χ_{het} [-]	$FE_{C_2H_4}$ [-]	V_{cell} [V]	χ_{hom} [-]
Jouny et al.[S22]	0.50	0.90	2.00	0.00
This work	0.50	0.70[S24]	3.69	0.00

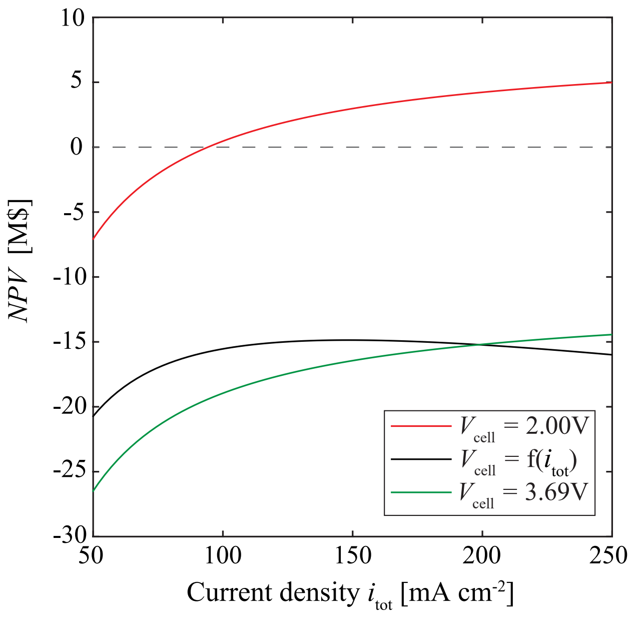

Figure S9: Comparison of the NPV for model M1 with the performance targets used by us for $V_{cell} = 3.69\,V$ (green line, reported in the bottom row of Table S6) and by Jouny et al.[S22] for $V_{cell} = 3.69\,V$ (red line, reported in the top row of Table S6) and a variable cell voltage (see Section S5, black line)

9 Summary of assumptions and their impact on the results

The aim of this work is to define a channel scale model which can sufficiently capture the interdependencies between the performance metrics (Faradaic efficiency, current density, cell voltage, heterogeneous conversion, and consumption) to study their influence on the different scales and economic outlook. The constraints for this model include a fast run time to make it suitable for optimisation studies and the limited available data from experimental results, which makes the validation and extraction of kinetic constants difficult.[S5] These constraints lead to the following assumptions discussed throughout the main manuscript and summarised below, together with their justification. Further, the foreseen impact of these assumptions on the NPV and the optimal operating conditions is discussed.

Assumptions of the *full* channel model M3

In this work only the cathode compartment of a GDE cell is modelled (see Section S3). The simplifications in regard of the geometry include that the gas diffusion layer of the cathode electrode is neglected. This layer adds an extra transport resistance for the gases to reach and leave the catalyst layer, since the purpose of this layer is to facilitate their transport. The work by Weng et al.[S15] shows that the influence on local concentration in the catalyst layer is minimal, justifying that we neglected this layer in this work. Further, species transport is only explicitly modelled in the gas channel, the cathode catalyst layer, and the liquid boundary layer. The extent of the liquid boundary layer is approximated through the Lévêque approximation[S25] (Eq. 7, main manuscript). The transport in the membrane, the electrolyte flow channels, and at the anode are not modelled. The ohmic resistance of the catholyte chamber and membrane as well as the expected overpotential at the anode are approximated through Ohm's law and the *hyperbolic sine approximation*[S20] (Eq. S21), respectively. Transport in the gas channel in the axial direction is dominated by convection. Pressure loss is expected to be small and its effect on flow velocity is therefore

neglected. It is further assumed that the chosen catalyst only produces ethylene from CO_2 or hydrogen from water. However, a catalyst which can selectively produce ethylene is so far not known. The most common catalyst used to produce higher hydrocarbons via electrochemical CO_2 reduction is copper, which produces a variety of gas and liquid CO_2 reduction products. Including a specific catalyst in the channel scale model, requires the kinetics of all products to be described by the Butler-Volmer equation (Eq. 12) with all individual kinetics constants. Nevertheless, most of the kinetic constants reported in literature vary significantly between different studies and reactor designs as it is difficult to decouple them from the mass transfer and local reaction conditions in the reactor.[S11] Therefore the reaction kinetics in this work are simplified with the only carbonaceous product being ethylene and therefore neglecting any additional gas and liquid products forming from CO_2. The catalyst layer is assumed to be fully flooded, while stability issues like gas breakthrough due to a pressure difference between the gas and liquid channel[S26] are neglected. Transport in the liquid phase is therefore mainly given by diffusion. Diffusion is further corrected for the porosity of the catalyst layer. The phase-transfer occurs at the gas-liquid boundary at the gas channel side. In the catalyst layer the electrochemical reaction and the homogeneous carbonate buffer reaction take place and act as a sink term for the CO_2. It is assumed that the reactor is constantly fed with fresh 1M $KHCO_3$ electrolyte, while recycling and post-treatment of the electrolyte is not considered in the costs. Further, migration of bicarbonate/carbonate ions is neglected[S4,S15] as only the cathode compartment is modelled. The resulting carbon balance from these assumptions is shown in Figure S10 a). The CO_2 lost to the carbonate buffer reaction is not recovered. Hence an increase in bicarbonate/carbonate formation leads to a decrease in the NPV.

Impact of assumptions on results

In comparison to the carbon balance resulting from the modelling assumptions (Figure S10 a)) the expected carbon balance for GDE reactors with a fixed type of membrane and anolyte are shown in Figure S10 b) and c). For a copper catalyst at the cathode various gaseous

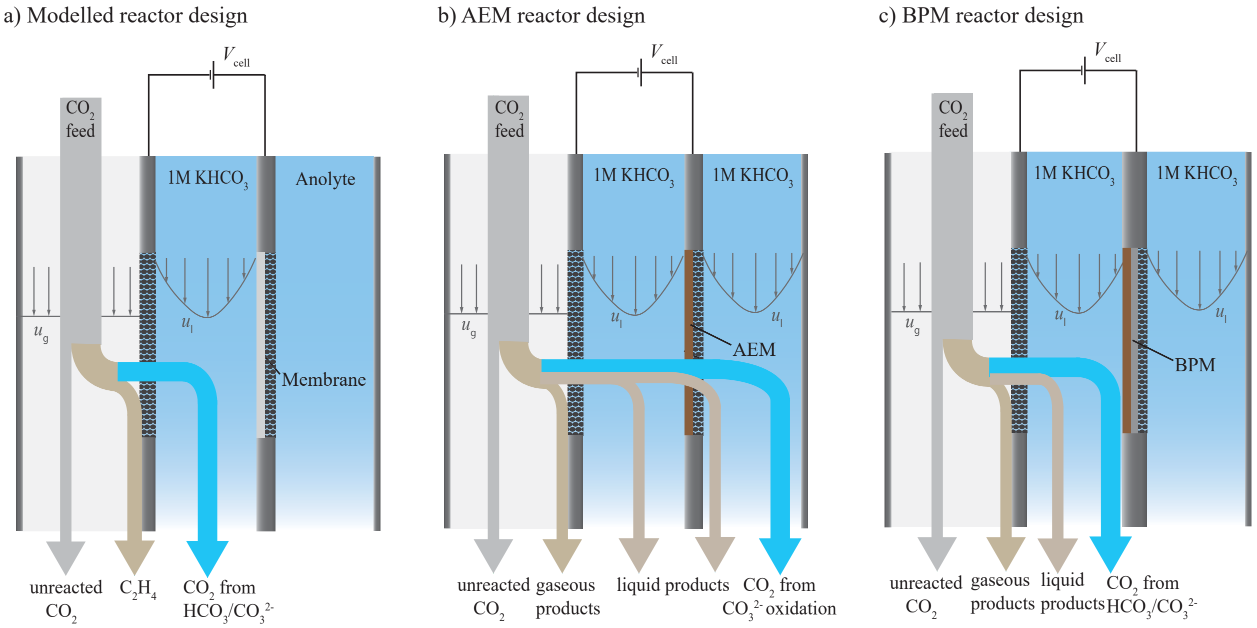

Figure S10: Carbon balance for the modelled a), an anion exchange membrane (AEM) b), and a bipolar membrane (BPM) c) GDE reactor.

and liquid carbonaceous products are expected to form. Considering additional gaseous products increases the required post-treatment steps for the cathode gas stream to separate the products, which adds additional investment and operating costs. It is expected that the profit from these products does not balance the additional costs therefore leading to a decrease of the overall *NPV*. Considering the effect of gas composition and the effect on separation efficiency further leads to higher optimal conversion rates compared to our study. The electrolytes are commonly recirculated, therefore liquid products need to be separated from the liquid streams to avoid accumulation. For anion exchange membrane (AEM) reactors up to 30 - 40% of the liquid products can crossover the membrane to the anode side and be oxidised.[S27] The effect of oxidation on the overall cell potential is generally low, therefore no changes in the predicted optimal current density and gas flow rate are expected. The AEM further allows for bicarbonate and carbonate ions to migrate to the anode side. It was found that under steady-state conditions the catholyte pH becomes alkaline and the main charge carrier is carbonate.[S28] The carbonate ions which migrate from the cathode to the anode then react in the liquid anolyte with the H^+ ions from the water

splitting reaction. This leads to the formation of CO_2 gas in the anolyte. Considering this effect the loss of CO_2 is expected to increase.[S29] An increase in the consumption rate of CO_2 intensifies the trade-offs shown between model M2 and M3 and shifts the optimal performance to lower CO_2 conversions. One approach to minimise liquid product and carbonate crossover to the anode is the use of bipolar membranes.[S30,S31] However for these membrane types the ohmic loss and hence the overall cell voltage increase. An increase in cell voltage leads to a decrease of the overall NPV as discussed in Section S8.

Overall it is expected that the absolute values for the NPV decrease if the required additional cleaning steps for the gas and liquid streams are considered. Further, the optimal operating conditions are expected to vary when considering a variety of products or additional loss terms for CO_2 such as degassing due to migration. However, the presented trade-offs and sensitivity towards the process economics are expected to be similar. Including the channel scale model into existing process models which consider additional pre- and post-treatment units presents an interesting next step and could for example give clarity under which conditions the recovery of the degassed CO_2 is desirable.

References

(S1) Subramanian, S.; Middelkoop, J.; Burdyny, T. Spatial reactant distribution in CO_2 electrolysis: balancing CO_2 utilization and faradaic efficiency. *Sustainable Energy Fuels* **2021**, *5*, 6040–6048.

(S2) Sisler, J.; Khan, S.; Ip, A. H.; Schreiber, M. W.; Jaffer, S. A.; Bobicki, E. R.; Dinh, C.-T.; Sargent, E. H. Ethylene electrosynthesis: A comparative techno-economic analysis of alkaline vs membrane electrode assembly vs CO_2–CO–C_2H_4 tandems. *ACS Energy Letters* **2021**, *6*, 997–1002.

(S3) Tan, Y. C.; Lee, K. B.; Song, H.; Oh, J. Modulating local CO_2 concentration as a general strategy for enhancing C-C coupling in CO_2 electroreduction. *Joule* **2020**, *4*, 1104–1120.

(S4) Kas, R.; Star, A. G.; Yang, K.; Van Cleve, T.; Neyerlin, K. C.; Smith, W. A. Along the channel gradients impact on the spatioactivity of gas diffusion electrodes at high conversions during CO_2 electroreduction. *ACS Sustainable Chemistry & Engineering* **2021**, *9*, 1286–1296.

(S5) Brée, L. C.; Wessling, M.; Mitsos, A. Modular modeling of electrochemical reactors: Comparison of CO_2-electolyzers. *Computers and Chemical Engineering* **2020**, *139*.

(S6) Hills, E. E.; Abraham, M. H.; Hersey, A.; Bevan, C. D. Diffusion coefficients in ethanol and in water at 298K: Linear free energy relationships. *Fluid Phase Equilibria* **2011**, *303*, 45–55.

(S7) Linstrom, P. NIST Chemistry WebBook, NIST Standard Reference Database 69. 1997.

(S8) Haynes, W. *CRC Handbook of Chemistry and Physics*, 95th ed.; CRC Press: Boca Raton, FL, 2014.

(S9) Damjanovic, A.; Dey, A.; Bockris, J. Kinetics of oxygen evolution and dissolution on platinum electrodes. *Electrochimica Acta* **1966**, *11*, 791–814.

(S10) Deen, W. *Analysis of Transport Phenomena*; Topics in Chemical Engineering; Oxford University Press USA: New York City, 1998.

(S11) Corpus, K. R. M.; Bui, A. M., Justin C.and Limaye; Pant, L. M.; Manthiram, K.; Weber, A. Z.; Bell, A. T. Beyond Tafel Analysis for Electrochemical CO_2 Reduction. *ChemRxiv* **2022**,

(S12) Schulz, K.; Riebesell, U.; Rost, B.; Thoms, S.; Zeebe, R. Determination of the rate constants for the carbon dioxide to bicarbonate inter-conversion in pH-buffered seawater systems. *Marine Chemistry* **2006**, *100*, 53–65.

(S13) Millero, F. J.; Graham, T. B.; Huang, F.; Bustos-Serrano, H.; Pierrot, D. Dissociation constants of carbonic acid in seawater as a function of salinity and temperature. *Marine Chemistry* **2006**, *100*, 80–94.

(S14) Yang, Z.; Li, D.; Xing, L.; Xiang, H.; Xuan, J.; Cheng, S.; Yu, E. H.; Yang, A. Modeling and upscaling analysis of gas diffusion electrode-based electrochemical carbon dioxide reduction systems. *ACS Sustainable Chemistry & Engineering* **2021**, *9*, 351–361.

(S15) Weng, L.-C.; Bell, A. T.; Weber, A. Z. Modeling gas-diffusion electrodes for CO_2 reduction. *Phys. Chem. Chem. Phys.* **2018**, *20*, 16973–16984.

(S16) Choi, B.-U.; Tan, Y. C.; Song, H.; Lee, K. B.; Oh, J. System Design Considerations for Enhancing Electroproduction of Formate from Simulated Flue Gas. *ACS Sustainable Chemistry & Engineering* **2021**, *9*, 2348–2357.

(S17) Dragnea, P.; Bildea, C. Process optimization of a butane-butene alkylation plant. *UPB Scientific Bulletin, Series B: Chemistry and Materials Science* **2014**, *76*.

(S18) Wiheeb, A. D.; Helwani, Z.; Kim, J.; Othman, M. R. Pressure Swing Adsorption Technologies for Carbon Dioxide Capture. *Separation & Purification Reviews* **2015**, *45*, 108–121.

(S19) Yang, Z.; Li, D.; Xing, L.; Xiang, H.; Xuan, J.; Cheng, S.; Yu, E. H.; Yang, A. Modeling and Upscaling Analysis of Gas Diffusion Electrode-Based Electrochemical Carbon Dioxide Reduction Systems. *ACS Sustainable Chemistry & Engineering* **2020**, *9*, 351–361.

(S20) Noren, D.; Hoffman, M. Clarifying the Butler–Volmer equation and related approximations for calculating activation losses in solid oxide fuel cell models. *Journal of Power Sources* **2005**, *152*, 175–181.

(S21) Verma, S.; Kim, B.; Jhong, H.-R.; Ma, S.; Kenis, P. J. A. A gross-margin model for defining technoeconomic benchmarks in the electroreduction of CO_2. *ChemSusChem* **2016**, *9*, 1972–1979.

(S22) Jouny, M.; Luc, W.; Jiao, F. General techno-economic analysis of CO_2 electrolysis systems. *Industrial and Engineering Chemistry Research* **2018**, *57*, 2165–2177.

(S23) Kibria, M. G.; Edwards, J. P.; Gabardo, C. M.; Dinh, C.-T.; Seifitokaldani, A.; Sinton, D.; Sargent, E. H. Electrochemical CO_2 reduction into chemical feedstocks: From mechanistic electrocatalysis models to system design. *Advanced Materials* **2019**, *31*, 1807166.

(S24) Dinh, C.-T.; Burdyny, T.; Kibria, M. G.; Seifitokaldani, A.; Gabardo, C. M.; García de Arquer, F. P.; Kiani, A.; Edwards, J. P.; De Luna, P.; Bushuyev, O. S.; Zou, C.; Quintero-Bermudez, R.; Pang, Y.; Sinton, D.; Sargent, E. H. CO_2 electroreduction to ethylene via hydroxide-mediated copper catalysis at an abrupt interface. *Science* **2018**, *360*, 783–787.

(S25) Lévêque, A. *Les Lois de la transmission de chaleur par convection*; Dunod, 1928.

(S26) Baumgartner, L. M.; Koopman, C. I.; Forner-Cuenca, A.; Vermaas, D. A. Narrow Pressure Stability Window of Gas Diffusion Electrodes Limits the Scale-Up of CO_2 Electrolyzers. *ACS Sustainable Chemistry & Engineering* **2022**, *10*, 4683–4693.

(S27) Zhang, J.; Luo, W.; Züttel, A. Crossover of liquid products from electrochemical CO_2 reduction through gas diffusion electrode and anion exchange membrane. *Journal of Catalysis* **2020**, *385*, 140–145.

(S28) Ma, M.; Clark, E. L.; Therkildsen, K. T.; Dalsgaard, S.; Chorkendorff, I.; Seger, B. Insights into the carbon balance for CO_2 electroreduction on Cu using gas diffusion electrode reactor designs. *Energy & Environmental Science* **2020**, *13*, 977–985.

(S29) Rabinowitz, J.; Kanan, M. The future of low-temperature carbon dioxide electrolysis depends on solving one basic problem. *Nature Communications* **2020**, *11*, 5231.

(S30) Wang, N.; Miao, R. K.; Lee, G.; Vomiero, A.; Sinton, D.; Ip, A. H.; Liang, H.; Sargent, E. H. Suppressing the liquid product crossover in electrochemical CO_2 reduction. *SmartMat* **2021**, *2*, 12–16.

(S31) Ma, M.; Kim, S.; Chorkendorff, I.; Seger, B. Role of ion-selective membranes in the carbon balance for CO_2 electroreduction via gas diffusion electrode reactor designs. *Chemical Science* **2020**, *11*, 8854–8861.
